# Mechanisms of Psychedelic Metaplasticity: Intracellular Receptor Pharmacology, Epigenomic Priming and Candidate Substrates of Persistence—A Narrative Review

**DOI:** 10.3390/ijms272110010

**Published:** 2026-11-08

**Authors:** Ivan Anchesi, Veronica Argento, Alessia Floramo, Maria Francesca Astorino, Sara Spinelli, Marco Calabrò, Giovanni Luca Cipriano

**Affiliations:** 1 IRCCS Centro Neurolesi “Bonino-Pulejo”, Via Provinciale Palermo, Contrada Casazza, 98124 Messina, Italy; ivan.anchesi@irccsme.it (I.A.); veronica.argento@irccsme.it (V.A.); alessia.floramo@irccsme.it (A.F.); giovanniluca.cipriano@irccsme.it (G.L.C.); 2 Department of Biomedical and Dental Sciences and Morpho Functional Imaging, University of Messina, 98125 Messina, Italy; mastorino@unime.it (M.F.A.); mcalabro@unime.it (M.C.)

**Keywords:** 5-HT2A receptor, psychedelics, metaplasticity, enhancer priming, H3K27ac, neuro-immune gatekeeping

## Abstract

A single dose of a serotonergic psychedelic produces benefits that persist for weeks beyond drug elimination, yet the substrate holding that capacity across the days-to-weeks consolidation window is undefined. Psychedelics are described as metaplastogens, agents that durably modulate the capacity for subsequent plasticity rather than sustaining a plastic state. This review assembles the candidate substrates and grades the evidence as established, indirectly supported or hypothetical. Intracellular serotonin 2A (5-HT2A) receptor pools and neurotrophin convergence are reviewed. Cortical histone H3 lysine 27 acetylation (H3K27ac) data show a persistent bidirectional change at seven days: elevated elements enriched for rhythmic processes alongside depotentiated enhancers annotated to synaptic plasticity. Because acetylation turns over within minutes to hours where it has been measured—a premise untested at these elements—we propose, as a framework rather than a demonstrated mechanism, that the signal reflects a displaced regulatory set-point, not a retained mark. The evidence base is narrow: one compound, frontal cortex of adult male mice, bulk neuronal nuclei, nothing beyond seven days and no causal test. Claims of persistence over weeks therefore concern the behavioural phenotype, not the chromatin state. The review closes with six falsifiable criteria and a matrix discriminating competing accounts.

## 1. Introduction

Modern psychiatry and translational neurology are undergoing a resurgence in mechanistic interest in psychedelic compounds as candidate therapies for brain disorders. Pathologies characterised by early synaptopathy and progressive cognitive decline, such as Alzheimer’s and Parkinson’s disease, display a synaptic vulnerability that may be counteracted by the stimulation of neuronal plasticity and cellular survival elicited by compounds such as psilocybin or lysergic acid diethylamide (LSD) [1,2,3,4]. The most striking property of these compounds, however, is not the rapidity of their onset but the persistence of behavioural benefit well beyond drug clearance [3]. This temporal dissociation defines what we call the “persistence paradox”: a single exposure produces sustained effects on the circuits of mood, fear and reward, yet the pharmacological stimulus is gone within hours. Although psychedelics plausibly reverse prefrontal cortex (PFC) atrophy through rapid spinogenesis [3,5,6], acute signalling cascades alone cannot account for the maintenance of that plasticity window. The mechanism required is therefore constrained in time before it is constrained in chemistry (Section 2.1).

This narrative review assembles the candidate substrates of psychedelic metaplasticity, grades the evidence available for each, and collects the observations that discriminate between them. For each candidate it sets out what the candidate requires, which of those requirements are currently unmet, and which of the resulting tests can be performed with established methods. The selection procedure, the uneven coverage it produces and the treatment of preprints are set out in the section Scope and Literature Selection.

The serotonergic system, and specifically the serotonin 2A receptor (5-HT2A), constitutes the molecular entry point for the effects of serotonergic psychedelics [1]. Conditional ablation of 5-HT2A receptors in the medial frontal cortex abolishes both the structural plasticity and the sustained behavioural action of psilocybin [7], and the relationship between this requirement and the parallel, tropomyosin receptor kinase B (TrkB)-dependent route described below is treated explicitly in Section 3.5, because the two literatures have often been read as contradictory. A pharmacological paradox nonetheless persists: serotonin (5-HT), the high-affinity endogenous agonist, evokes neither the perceptual effects nor the profound structural plasticity characteristic of psychedelics [3,8].

This divergence is generally attributed to two non-exclusive principles: functional selectivity (biased agonism) and subcellular location bias. Unlike serotonin, which is polar and membrane-impermeant, the lipophilic psychedelics gain direct access to intracellular pools of 5-HT2A receptors, including those resident in the Golgi apparatus [9]. Activation of this internal receptor pool, together with signalling organised by the postsynaptic density protein 95 (PSD-95), is thought to be required for the changes in gene expression and chromatin architecture that support long-term plasticity [10,11,12,13,14].

One account that can be assembled from these literatures, and the one examined in most detail here, is enhancer latching: a persistent shift in the cis-regulatory set-point of plasticity-related genes, with the enhancer landscape displaced bidirectionally and relaxing toward baseline over days to weeks. The term latching is used throughout for that displaced two-sided state—one set of elements elevated, another depressed, relaxing together toward baseline—and is adopted here as a descriptive label for the published signature rather than as a mechanism. The review then examines the candidate maintenance mechanisms against physical timescales, treats the glial permissive microenvironment as an enabling condition rather than a required step, and collects the empirical benchmarks that would distinguish chromatin-mediated metaplasticity from the competing accounts in the field, including ordinary experience-dependent consolidation. Evidence is graded explicitly at each level, and findings resting on preprints or on single preclinical reports are flagged at every mention.

### Scope and Literature Selection

This is a narrative review with deliberately uneven coverage, and the selection procedure is stated so that readers can judge its reach. Studies were identified in PubMed/MEDLINE and Google Scholar, supplemented by the reference lists of the retrieved articles and by the bioRxiv preprint server, searched from inception to September 2026. Search terms combined compound and receptor terms (psychedelic, psilocybin, LSD, 2,5-dimethoxy-4-iodoamphetamine (DOI), *N*,*N*-dimethyltryptamine, 5-HT2A, serotonin 2A receptor) with mechanism terms (metaplasticity, neuroplasticity, enhancer, chromatin, histone H3 lysine 27 acetylation (H3K27ac), histone acetylation, persistence, dendritic spine, TrkB, glycogen synthase kinase 3β (GSK3β), neuroinflammation), using the Boolean operators AND and OR. Inclusion followed the review’s question rather than a formal protocol: reports bearing on persistence beyond 48 h after a single exposure were included irrespective of outcome and are covered in full, since that body of literature is small, whereas the acute signalling literature is extensive and was sampled for the specific evidential thread each report contributes rather than comprehensively. Chronic dosing paradigms, non-mammalian systems and clinical outcome studies without a molecular readout are cited only where they bound the generalisability of a mechanistic claim. Preprints are cited only where no peer-reviewed equivalent exists, are identified as preprints at every mention, and no prediction in Section 2.5 depends on one. Where findings conflict—the TrkB-binding controversy of Section 3.5 is the principal instance—both sides are cited and the conflict is analysed rather than resolved by selection. Because the procedure was not systematic, selection bias cannot be excluded, and this review should be read as an argued synthesis rather than as an exhaustive survey.

## 2. The Persistence Paradox and the Candidate Substrates of Persistence

### 2.1. The Empirical Shape of the Problem

Preclinical work and emerging clinical evidence indicate that psychedelic treatment produces measurable therapeutic effects within hours of administration [15], while the pharmacokinetic residence of the compound is on the order of a single day. Resolving this dissociation requires identifying a biologically stable substrate that operates as a “molecular memory” downstream of, and independent from, drug occupancy. Precedent for such temporal decoupling comes from rapid-acting antidepressants, where a single administration engages mechanisms including GSK3β inhibition and histone deacetylase (HDAC) modulation [16,17] that yield structural neuroplasticity and behavioural effects persisting for days to weeks [2,18].

The paradox imposes a constraint before it admits any candidate, and the constraint is temporal rather than molecular. Whatever holds the state must relax on the timescale of the phenomenon: slower than kinase signalling, more stable than messenger RNA, and yet reversible enough to remain physiological. We therefore organise the candidates by characteristic time constant rather than by molecular class.

The criterion must be applied to carriers rather than to molecular classes. The time constant of a network is not the time constant of its components: a configuration can persist while every molecule composing it is replaced, which is the ordinary situation in a post-mitotic cell and is precisely what Section 4.3 will require. What the criterion excludes is therefore the claim that a given molecule itself carries the state, not the possibility that a system assembled from fast components does so.

On that reading three carriers fail, and one failure is more consequential than the others. Individual kinase signalling events operate over seconds to minutes and are spent before the phenomenon begins. Individual messenger RNA molecules turn over within hours, and here the argument is reinforced by measurement, since transcripts for key synaptic genes return toward baseline while the behavioural and structural phenotype persists [11,19]—though, as we note below, that measurement was made in bulk and the exclusion is correspondingly weaker than it appears. Individual acetyl groups turn over within minutes to hours [20,21,22], and this applies with full force to the very mark on which the enhancer-latching account rests: it is improbable that any acetyl group deposited at the time of exposure is present seven days later. Two qualifications are owed to a premise this central. The measurements establishing rapid turnover were made largely in dividing non-neuronal cells and by bulk labelling [20,21], and the same work that establishes rapid average turnover also identifies a minority of acetylation sites with substantially slower kinetics [20]; no measurement of acetyl turnover exists for the persistent enhancer set in post-mitotic cortical neurons. The premise is therefore a strong prior imported from other systems rather than a measurement in this one, and the shape of any acetylation-indexed account depends on it: were the persistent set to fall among the slow-turnover minority, no set-point reformulation would be needed and the simpler retained-mark account would be available. That the central observable fails as a molecular carrier is not an incidental difficulty. It is the constraint that determines what form such an account can take, and it converts the central question from which molecule survives into which state variable or feedback dynamic conserves the difference while the molecules are replaced. Section 4.3 compiles the candidate answers; none of them is currently established.

Several candidates fall inside the window. Scaffold proteins of postsynaptic density have lifetimes measured in days to weeks [23], so a long-lived, palmitoylation-stabilised pool could hold an altered synaptic state with no nuclear contribution at all. The palmitate cycle on PSD-95 is itself activity-regulated and reversible on a timescale of minutes to hours [24,25], so the persistence of such a pool is a quantity to be demonstrated rather than assumed. A third structural possibility is more demanding still: the spines formed after the drug may themselves be the store, since selective optical ablation of antidepressant-induced spines abolishes the maintained behavioural effect while leaving the acute response intact [26]. That is the causal design the enhancer-latching account asks for in Test 2, already executed on a competing substrate, and it is returned to in Section 7. Nucleosome exchange rates, local acetyl-CoA availability, perineuronal extracellular matrix (ECM) remodelling and circadian gating all have characteristic times in the days-to-weeks range; each is examined in Section 4.3. DNA methylation belongs here too. Neuronal methylation does not have a single characteristic timescale: rapid, experience-driven changes accompany memory formation [27,28], so ruling it out as too slow both misstates the biology and contradicts the distinction between molecules and state variables drawn immediately above. It remains among the alternatives, and what matters is its dynamics at the loci actually in question rather than the average behaviour of the modification. Histone monoaminylation belongs here as well: transglutaminase 2 transfers serotonin to glutamine 5 of histone H3, histone H3 glutamine 5 serotonylation (H3Q5ser) is combinatorially coupled to histone H3 lysine 4 trimethylation (H3K4me3) and enhances transcription factor II D (TFIID) binding [29], and the analogous dopaminylation mark in the ventral tegmental area is dynamically regulated across weeks and causally controls drug seeking [30]. It is examined alongside the other candidates in Section 4.3.

Two of the candidates inside the window are routinely set aside on grounds that do not survive examination, and both therefore remain live alternatives to the account developed here.

Sustained transcription is the first candidate. It is commonly treated as excluded because messenger RNA levels for key synaptic genes return to baseline [11], while the behavioural and structural phenotype persists [19]. That inference is sound for the bulk tissue in which those measurements were made, but it does not exclude sustained low-amplitude transcription confined to a sparse population. The point is not hypothetical: the durable behavioural effect of psilocybin has been localised to pyramidal tract neurons specifically, since silencing them, but not intratelencephalic neurons, eliminates it [7]. A transcriptional change restricted to that class would be diluted below detection in bulk measurements. The same limitation of cellular resolution is raised against the chromatin evidence in Section 4.2 and applies equally here. Sustained sparse transcription therefore remains a live competing account, and Section 7 states what would separate it from ours.

Proteostatic persistence is the second candidate. It is usually dismissed on the grounds that a stable protein pool cannot explain reactivation of transcriptional programmes weeks later. That objection assumes a requirement the chromatin accounts do not impose: none of them calls for transcriptional programmes to be reactivated at a distance, only for responsiveness to be altered, which is the sense in which a psychedelic is said to lower the cost of re-entering the plastic state. Once the requirement is stated correctly the objection loses its force because a long-lived scaffold pool can alter responsiveness on its own. Persistent synaptic-marker elevation after a single dose has been reported at the protein level (Section 4.4). PSD-95 stabilised at the membrane by palmitoylation is a plausible carrier of such persistence [24,25]. A synapse holding a larger, internalisation-resistant scaffold pool answers the same glutamatergic input differently, with no chromatin contribution required. Proteostatic persistence is therefore not a subordinate layer of a chromatin account but a genuine alternative to it, and it is treated as such in Section 4.4 and Section 7 rather than integrated as supporting evidence.

The remaining candidate is a change in the regulatory potential of chromatin that alters how the genome responds to subsequent physiological signals without requiring continuous transcriptional output [11]. It receives the most detailed treatment here, not because the alternatives are excluded—two of them are not—but because it is the candidate for which direct persistence data exist and the one that yields the most specific experimental criteria. The temporal criterion already constrains the form such an account can take: because acetylation itself does not meet that criterion, a persistent mark cannot be asserted, and what the data support is a persistent set-point, which is a different and more demanding proposition.

### 2.2. Boundaries of the Question: Which Timescale the Available Evidence Addresses

A boundary condition should be stated at the outset, because a framework of this kind is routinely miscast without one. Enhancer latching is proposed as the substrate of the consolidation window—days to weeks—and not as the store of multi-year clinical outcomes. The two are separated by several orders of magnitude and, on the available evidence, by a change of mechanism: the chromatin state does not carry the five-year effect, it permits the circuit- and behaviour-level reorganisation that does. On this view, durable clinical benefit of the kind documented in long-term follow-up of psilocybin-assisted therapy [31,32] is maintained by the same processes that maintain any consolidated learning, and the contribution of the drug is confined to opening and biasing the window in which that learning is acquired.

**Figure 1:**
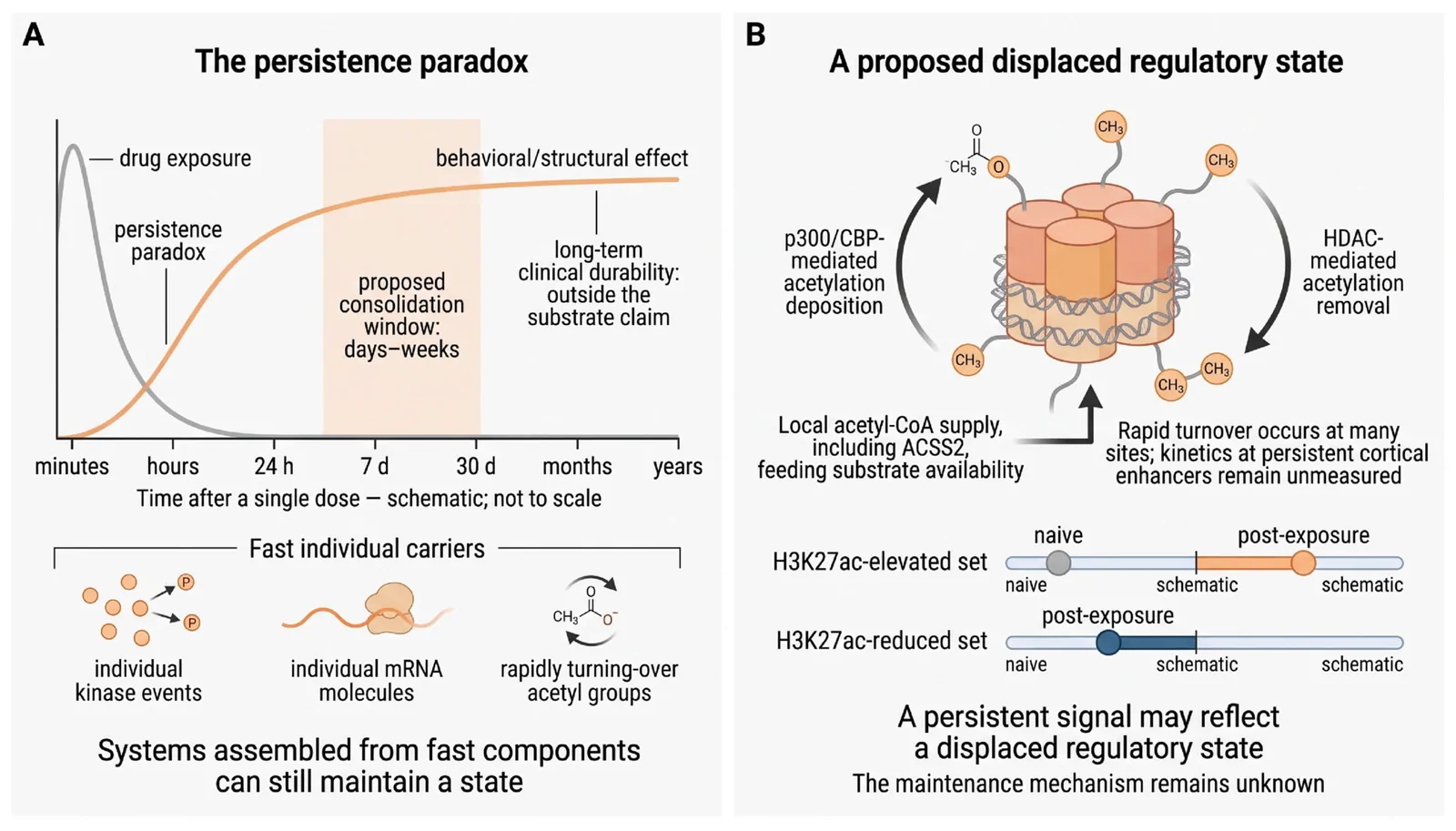
The persistence paradox and the proposed regulatory set-point account. (**A**) Schematic temporal dissociation between drug exposure and longer-lasting behavioural or structural effects after a single psychedelic dose. The shaded region represents the proposed days-to-weeks consolidation window addressed by this review; multi-year clinical outcomes do not establish persistence of the same molecular substrate. Individual kinase events, messenger RNA molecules and rapidly turning-over acetyl groups are insufficient as long-lived carriers, although systems assembled from such components can maintain a state. Curves and temporal spacing are illustrative rather than quantitative. (**B**) Histone acetylation reflects the balance between p300/CBP-mediated deposition, histone deacetylase (HDAC)-mediated removal and substrate availability, including local acetyl-CoA production by acetyl-CoA synthetase 2 (ACSS2). The sliders illustrate opposing directions of persistent H3K27ac change, without establishing functional priming or a fixed equilibrium. A displaced regulatory state is proposed to explain signal persistence despite molecular turnover. Acetylation kinetics at the persistent cortical enhancer set and the mechanism maintaining its displacement remain unknown [11,20,21,22,36]. Both panels are conceptual schematics and show no measured data; the temporal relationships illustrated in (**A**) are assembled from behavioural, structural and chromatin studies that used different compounds, species and endpoints, and do not derive from a single experiment. CBP—CREB-binding protein; HDAC—histone deacetylase; ACSS2—acetyl-CoA synthetase 2; H3K27ac—histone H3 lysine 27 acetylation. Created with BioRender, Cipriano, G. (2026) https://BioRender.com/39a8kax, accessed on 8 October 2026.

If clinical durability data can neither confirm nor exclude a substrate claim of this kind, then every test of it is a molecular measurement made inside a seven-to-thirty-day window, and—as Section 8 sets out—those measurements are currently available only in rodents. No substrate-level test is therefore available in humans at present. The gap is narrower than that makes it sound, because the phenomenon and the substrate can be addressed separately. A persistent human marker exists—hippocampal–cortical desynchronisation measurable weeks after a single dose [33]—and human paradigms exist that measure the capacity to induce new plasticity rather than a benefit already acquired, whether as long-term potentiation (LTP)-like visual cortical plasticity [34] or as cognitive flexibility after treatment [35]. These can corroborate or exclude the phenomenon itself; they cannot address the configuration proposed as its substrate. The two questions should be held apart when the near-term utility of this literature is assessed. The temporal constraint and the proposed distinction between molecular turnover and the persistence of a regulatory configuration are summarised in Figure 1.

### 2.3. Relaxation with Altered Responsiveness, and the Question of True Hysteresis

The published time course is better described as a slow relaxation than as a fixed displacement. The enhancer landscape does not remain pinned at a displaced position; it returns toward baseline with a time constant of days to weeks rather than hours. What defines the state is not stasis but lag: during the return, the same physiological stimulus elicits a different transcriptional response than it would in a drug-naive animal.

That behaviour is often described as hysteresis. The term makes a stronger commitment than the data support. In its technical sense it denotes path dependence in a bistable system (Box 1), whereas slow relaxation with altered responsiveness is a weaker and more common phenomenon, and it is all the data require. The state is therefore described here as a displaced set-point undergoing slow relaxation, with true bistability as an additional and separately testable proposition. Its signatures would be distinctive: a threshold rather than graded dependence on dose, an all-or-none rather than continuous distribution of the persistent state across single cells, and dependence on prior exposure history at matched current dose. None of these has been examined.

Box 1 **Terms used in this review. The six terms below are used in the senses defined here throughout.**

Metaplasticity—a change in the capacity for subsequent plasticity, rather than a continuously sustained plastic state, in the sense in which Abraham and Bear introduced the term (Section 2.4).

Metaplastogen—an agent producing such a change; the term is used as it was introduced for rapid-acting antidepressants and carries no commitment to a substrate.

Enhancer latching—the descriptive label used here for the published seven-day signature: a bidirectional displacement of the cortical H3K27ac landscape, one set of elements elevated and another depressed, relaxing together toward baseline. It names an observation together with a proposed reading of it, and not a demonstrated mechanism.

Regulatory set-point—the equilibrium between p300/CREB-binding protein (CBP)-mediated deposition, histone deacetylase (HDAC)-mediated removal and local acetyl-CoA availability at a given element. A displaced set-point denotes persistence of that equilibrium position while the individual acetyl groups turn over.

Priming—a lowered stimulus requirement for inducing a given transcriptional or synaptic response. It is a claim about re-inducibility, must be measured as a stimulus–response relationship, and cannot be read off the sign of a histone mark.

Slow relaxation versus hysteresis—the data support slow relaxation with altered responsiveness: the landscape returns toward baseline with a time constant of days to weeks, while the same stimulus elicits a different response during the return. Hysteresis, in the technical sense, requires bistability and path dependence, with two states available at the same input; it is used in this review only where that stronger claim is explicitly at issue, and its signatures—dose threshold, all-or-none single-cell distribution, and dependence on prior exposure history—have not been examined.

Framing the substrate as a slow relaxation carries three consequences. It removes the requirement for a permanently self-sustaining mark, which no chromatin modification in a post-mitotic neuron can plausibly supply; it implies a specific temporal shape, discussed in Section 4.1, in which some enhancer sets decay measurably between 48 h and seven days while others do not; and it makes the therapeutic window finite and measurable, with implications for the timing of psychological integration, which on this reading falls inside the lag rather than after it.

An account that accommodates both a plateau and a decay is not decidable, so the relevant time constant has to be bounded before it is measured, and the bounds follow from the phenomenon rather than from a preference. A dominant time constant for the persistent enhancer set in the range of approximately 7 to 60 days is the only range compatible with a substrate of the consolidation window: a measured relaxation faster than roughly 48 h would place the substrate inside the acute pharmacological response and leave the persistence unexplained, while a relaxation slower than roughly six months would approach the multi-year regime excluded in Section 2.2. A time course that returned either outcome would therefore exclude a set-point account of the consolidation window, and this is the first quantity a future study should report.

Nothing in the available data licenses this range, and it is important not to present it as though it did. The existing time course ends at seven days, and the persistently elevated set is still rising at that point rather than relaxing (Section 4.1), so its decay has not been observed at all. Duration of observation, time to peak, and decay constant are three different quantities and only the first two have been sampled. Estimating the third requires timepoints beyond seven days, which do not exist for this measurement; the range stated above is a design requirement for that experiment, not a result derived from the existing data.

A terminological clarification is warranted, because it bears directly on the mechanistic claim. In canonical chromatin nomenclature, histone H3 lysine 27 acetylation (H3K27ac) demarcates active enhancers and is used precisely to distinguish them from poised and inactive elements [37]. That convention requires its own qualification, because every acetylation-indexed account depends on it. This is the strongest available objection to an acetylation-indexed account, and the term indicator is used here in its light rather than in spite of it. The term enhancer is likewise preferred to epigenetic throughout, since neurons are post-mitotic and the marks at issue are neither self-templating nor mitotically heritable; “epigenetic” in the strict sense does not apply, and the narrower term states the claim more accurately.

An instructive example of decoupling is the capacity of psilocybin to rescue cognitive deficits for weeks without maintaining elevated messenger RNA levels of key synaptic genes such as *Dlg4* (PSD-95) [19]. Mechanistically, latching would lower the effective activation threshold of regulatory elements, enabling faster and amplified re-engagement of activity-dependent transcriptional programmes in response to endogenous signals long after pharmacological clearance. The proposition at issue is therefore specific and testable: the psychedelic does not maintain the plastic state; it lowers the cost of re-entering it.

### 2.4. Position Relative to Existing Accounts

It is important to situate this proposal within an existing conceptual frame rather than alongside it. The phenomenon under discussion—a stimulus that alters the capacity for subsequent plasticity rather than producing a continuously sustained plastic state—is metaplasticity in the sense in which Abraham and Bear introduced the term [38,39], and rapid-acting antidepressants, including psychedelics, have already been characterised as metaplastogens on precisely this basis [40]. A recent synthesis likewise identifies the reopening of critical periods, the induction of metaplasticity and the reorganisation of the extracellular matrix as unifying properties of psychedelic action [41]; the primary evidence for that account is the demonstration that 3,4-methylenedioxymethamphetamine (MDMA) reopens the oxytocin-dependent critical period for social reward learning [42] and that the duration of reopening tracks the duration of subjective drug action across psychedelic compounds [43]. What these accounts establish is the phenomenology: that the capacity for plasticity is durably altered, and that this is better captured by a learning model than by a biochemical imbalance model [41]. What they do not specify is the physical substrate in which the altered capacity resides between the drug exposure and its later expression. A persistent cis-regulatory configuration is the candidate for that substrate examined here.

Three distinct propositions are current in this literature and are easily run together; separating them determines what any given experiment can decide.

The first is causal and is the proposition on which the field’s evidence should be weighed: in a paradigm specified by compound, cell population and time window, a persistent cis-regulatory configuration contributes to the increased capacity to induce new plasticity, such that selective normalisation of that configuration reduces the increase while leaving ordinary plasticity intact. No published experiment addresses it. The configuration and the expected effect must be specified before the measurement, otherwise the proposition reduces to the untestable suggestion that chromatin might matter.

The second is stronger: that the configuration is not merely a necessary mediator but the store of the information—the place where the altered capacity resides between exposure and expression. Nothing reviewed here establishes it, and the experiments set out in Section 2.5 would not by themselves settle it, for reasons given in Section 4.3.

The third comprises the auxiliary mechanisms—the directional assignment of Section 4.2, the glial permissive branch of Section 5, and the circadian contribution of Section 4.3. These stand or fall independently of the first, and evidence against them does not bear on it.

Two consequences follow for how negative results in this area should be read. Every negative result is referred to the proposition actually tested: failure of an auxiliary mechanism is not evidence against the causal proposition, while failure of a candidate state—after verified normalisation and with demonstrated experimental sensitivity—rejects the version of the account that attributes the predicted contribution to that state. And because H3K27ac is an indicator rather than a definition, absence of a persistent acetylation signature does not exclude regulatory memory in some other form; it rejects the acetylation-based version of the proposal.

### 2.5. Criteria for Future Experimental Tests, with Directions Specified in Advance

An account that accommodates every outcome cannot be decided by experiment, and a prediction that admits either sign is not a prediction. The directions and criteria below are therefore specified in advance of the experiments, and are offered as design requirements for the studies the field still needs. Two preliminaries govern all six.

The first concerns what is being falsified. Persistent behavioural benefits, an altered capacity for new plasticity, and retention of something already learned are three different quantities, and the substrate question reviewed here concerns only the second. A drug that leaves an animal better off is not thereby shown to have altered its capacity to learn; an intervention that erases a modified capacity may leave an already-consolidated benefit entirely intact. It follows that behavioural benefit surviving a chromatin manipulation is not evidence against a chromatin substrate, because the quantity at issue is the capacity and not the consolidated outcome. The prediction below therefore takes metaplasticity—the response to a subsequent challenge—as the primary endpoint, and behavioural benefit only as a secondary one.

The second concerns where a change is located. A larger transcriptional response to a single stimulus can arise from greater excitability upstream of the nucleus rather than from a lowered enhancer threshold, and a single stimulus intensity cannot distinguish the two. Demonstrating a threshold change requires a stimulus–response relationship across intensities and localising it to the nucleus requires comparing conditions in which the intracellular input reaching the nucleus is itself comparable.

Test 1—Amplified re-induction. A physiological stimulus delivered days after a single psychedelic dose should elicit a larger and faster induction of plasticity genes than in a drug-naive animal. The test requires a stimulus–response curve rather than a single intensity, and the expected result is a leftward shift of that curve: a change in the stimulus required to reach a given response.

Two qualifications keep this prediction honest. A shifted transcriptional curve is not by itself metaplasticity, because transcriptional sensitisation and synaptic metaplasticity are not synonyms; the transcriptional measure must be paired with a direct measure of the capacity to induce new synaptic plasticity in the same preparation, and the proposition at issue is about the latter. And elevated basal transcription or increased somatic excitability do not automatically exclude a chromatin contribution—they can coexist with one. What they do is make the attribution less selective, since a shift could then be explained without invoking the enhancer landscape. Basal equivalence therefore defines the specific variant of the account in which priming occurs without sustained activation, rather than serving as a pass–fail criterion for any chromatin involvement.

Test 2—Causal contribution of persistent displacement. Selective normalisation of the candidate state should reduce the acquired increment in plastic capacity by the predicted magnitude, while leaving ordinary plasticity intact. This is the test that bears on the first proposition of Section 2.4. Complete abolition would be expected only under the stronger proposition, which assigns the configuration a role necessary for the entire increment; a selective reduction that is recoverable by rescue is a positive result for the core, not a failure of it.

Lowering acetylation globally, whether by degron-mediated depletion of p300/CBP or by broad deacetylase recruitment, is not a reset: applied to a bidirectional signature it would normalise the elevated set while depressing the already depressed set further, and p300/CBP is required for many ordinary responses, so losing its function is not equivalent to erasing a specific trace.

The correct form is a normalisation toward naive values that respects the sign of the alteration at each element—restoring the depressed set as well as the elevated one. The diagnostic outcome is a selective reduction of metaplasticity after the perturbation is withdrawn, with viability, basal responses and ordinary plastic capacity preserved, followed by recovery of the phenotype on targeted restoration of the state. Erasure without rescue is weak evidence, since almost any disruption can abolish a phenotype. We note too that preserving an acute response that occurred before the intervention is a weak control; what must be shown intact is the capacity to respond at the time of the second challenge. Section 4.3 retains complete erasure as the strongest experimental case, distinguished there from this minimum requirement.

Test 3—Directional assignment across the bidirectional set. If the direction of persistent change maps onto gene function, enhancers of genes that constrain or stabilise plasticity should lose activity and enhancers of genes that permit or induce it should retain or gain activity. The inverse assignment would exclude this directional reading, in the sense of the hierarchy set out in Section 2.4, and not the causal core. Two conditions must be met before this is testable. The classification must be fixed before the direction is examined, since otherwise the prediction is circular. But pre-registration does not by itself make a classification valid: assigning genes to permissive and constraining categories requires independent functional evidence and must be specific to cell type and context, because most synaptic genes act in both directions depending on circumstances. A pre-registered but biologically arbitrary list would produce a formally clean and substantively empty test. Standard ontologies do not supply the needed distinction (Section 4.2).

Test 4—Overlap with the acute response. The persistent set is sometimes treated as a subset of the acute response by construction, with absence of overlap taken to indicate non-causal drift. Neither follows. The clusters were derived by temporal clustering over the union of elements differential at one or more timepoints, which does not make late elements subsets of an early response; and a cascade causally initiated by the drug may legitimately recruit new regulatory elements at a later stage, so independence between early and late sets is not evidence against a causal link. What the comparison actually distinguishes is retention of the same elements from sequential regulatory recruitment. Its absence excludes the first variant only. Since the dataset contains no chromatin timepoint inside the acute window, the comparison cannot be made at all with existing data.

Test 5—Upstream position of neuro-immune permissiveness. If permissiveness is upstream of consolidation, immune manipulation during the days following administration should erase enhancer persistence without blocking the acute effect. A systemic endotoxin challenge is not an adequate test, since lipopolysaccharide alters sickness behaviour, hypothalamic–pituitary–adrenal activity, sleep architecture and brain-derived neurotrophic factor (BDNF) tone simultaneously. The test requires glia-restricted manipulation of nuclear factor κB (NF-κB) signalling, a factorial drug-by-manipulation design, the enhancer landscape rather than behaviour as the primary readout, challenge timing inside versus outside the window as an internal control, and comparison of inflamed with basal tissue. Transactivation rather than nuclear translocation must be assayed, for the reason given in Section 5.1. We also note that removing a constraint may permit a trace to be written without specifying its content, so a positive result establishes permissiveness and not instruction.

Test 6—Circadian dependence of maintenance. The persistently elevated set is enriched for rhythmic processes and the nuclear factor interleukin-3-regulated (NFIL3) motif (E2: motif enrichment only, occupancy unmeasured), which raises the possibility that circadian processes contribute to maintaining the displaced state. Testing this is harder than it appears, and the rate-versus-composition contrast does not work.

Three controls are prerequisites rather than refinement. H3K27ac itself oscillates across the day at enhancer elements, so sampling must resolve mean, phase and amplitude separately; a difference at a single clock time may be a phase shift. Treated and control animals receive the same light–dark cycle, so a circadian contribution requires demonstrating a difference in the response to that cycle—in oscillator phase, coupling or factor occupancy—rather than assuming the cycle acts differentially. Furthermore, motif enrichment must be replaced by measured NFIL3 occupancy before the factor is implicated at all. With those in place, the prediction is that disrupting the cycle during the window shortens the interval over which the altered capacity is expressed, without altering the acute drug response. Where the two conditions stated at the end of this section are met, the result eliminates a circadian contribution to maintenance; it says nothing about the other candidates in Section 4.3.

One comparison is necessary. Enhancer priming without concurrent transcriptional elevation, reorganisation of regulatory contacts, and differential response at recall have already been described in the hippocampal engram after contextual fear conditioning [44]. This supports the plausibility of the causal proposition, but it substantially reduces the discriminating power of its signatures: persistence, and dissociation from basal RNA, are not by themselves diagnostic of pharmacological metaplasticity because ordinary learning produces them too. A pharmacological account therefore requires that the configuration contribute causally to a modified capacity to respond to a subsequent experience, distinguishable from retention of the initial experience—which is why the falsification object stated at the head of this section is not a technicality but the condition under which any of these tests means anything.

A final preliminary concerns what is currently executable, because the six tests are not symmetrical in difficulty. Tests 1, 3, 5 and 6 can be attempted with established methods: stimulus–response curves paired with a synaptic measure, pre-registered functional classification, glia-restricted manipulation in a factorial design, and circadian disruption with mean, phase and amplitude separated. Test 2 cannot. As stated it requires a normalisation toward naive values that respects the sign of the alteration at each of thousands of elements, whereas targeted epigenome editing acts at a small number of selected loci [45]; the realisable experiment therefore perturbs a sampled subset of the persistent set, and a negative result obtained that way constrains the sampled loci rather than the configuration. Test 4 cannot be attempted at all with existing data, for the reason given above. The asymmetry is stated explicitly because a test that current technology cannot perform at the required scale belongs in a research programme rather than among the field’s near-term options.

Because Test 2 is realisable only on a sampled subset of the persistent elements, so the interpretation of its outcomes must be scoped in advance. A positive result at a sampled subset establishes a causal contribution of those elements and does not license a claim about the configuration as a whole. A negative result constrains the sampled loci only, and is uninformative unless the manipulation is confirmed to have normalised the targeted elements and the study excludes the predicted effect size with adequate precision; a merely non-significant outcome establishes nothing. The same two conditions govern the null results of Tests 5 and 6. Partial outcomes—reduction without abolition—are the expected result under the weaker causal proposition of Section 2.4 and should not be reported as failures of it.

Each of these tests is currently untested. Table 1 collects this material as a systematic framework for empirical testing, stating, layer by layer, the timescale proposed, the current evidentiary status and the principal outstanding test. The observations that separate this account from each competing substrate are set out in Section 7.

## 3. Triggering, Functional Selectivity and Signalling Architecture

**Figure 2:**
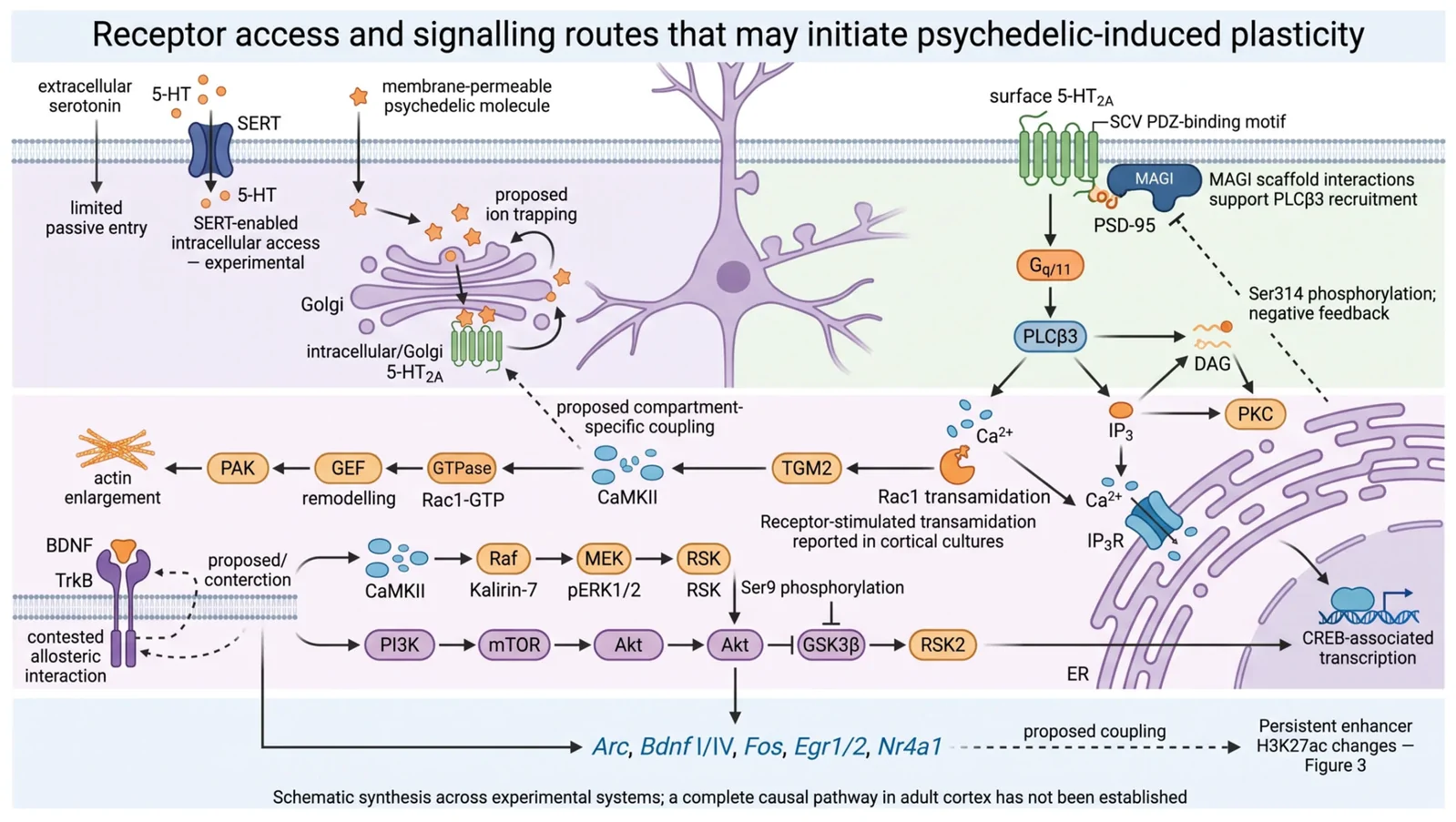
Receptor access and signalling routes that may initiate psychedelic-induced plasticity. Membrane-permeable psychedelics can engage intracellular 5-HT_2_A receptor pools; experimentally enabling serotonin access through the serotonin transporter (SERT) restores its plasticity-promoting activity [9]. At the cell surface, postsynaptic density protein 95 (PSD-95) and MAGI scaffold interactions organise receptor trafficking and phospholipase C-β3 (PLCβ3) signalling [10,47,79,80]. Calcium-dependent pathways engage local Kalirin-7–Rac1–PAK signalling involved in spine remodelling and nuclear ERK–CREB-associated transcription [12,48,49]. Receptor-stimulated Rac1 transamidation has been characterised in cortical cultures [46]; its preferential coupling to Golgi-resident receptors, and Golgi ion trapping, remain proposed. BDNF–TrkB signalling provides an additional route to plasticity, while direct psychedelic binding within the TrkB transmembrane dimer remains contested [2,51,93]. Selected convergence and feedback pathways are included. Solid connectors indicate reported signalling relationships, dashed connectors indicate proposed or contested links, and T-bars indicate inhibition. The schematic integrates findings from different experimental systems and does not establish a complete causal pathway in the adult cortex. Coupling these acute pathways to the persistent enhancer signature shown in Figure 3 remains untested. 5-HT2A—serotonin 2A receptor; 5-HT2B—serotonin 2B receptor; BDNF—brain-derived neurotrophic factor; CREB—cAMP response element-binding protein; ERK—extracellular signal-regulated kinase; LSD—lysergic acid diethylamide; MAGI—membrane-associated guanylate kinase with inverted orientation; PAK—p21-activated kinase; Rac1—Ras-related C3 botulinum toxin substrate 1; TGM2—transglutaminase 2; TrkB—tropomyosin receptor kinase B. The systems contributing to it differ: receptor-stimulated Rac1 transamidation was characterised in primary cortical cultures, the 5-HT2A–TrkB convergence principally in murine neural stem cells, MAGI/PLCβ3 coupling and the LSD-bound receptor structure in heterologous systems and in the 5-HT2B receptor, respectively. Intracellular receptor pools and 5-HT2A were required for durable effects in the adult mouse cortex. No single system supports the whole schematic, and the reference numbers in this legend follow the attributions made in the corresponding sections of the text. Created with BioRender, Cipriano, G. (2026) https://BioRender.com/39a8kax, accessed on 8 October 2026.

The biochemical trigger delivered by psychedelics at the 5-HT2A receptor is articulated through a layered signalling architecture whose components differ in subcellular localisation and in molecular partners. This section sets out that architecture, from the compartment in which the signal originates to the convergent hub at which pharmacologically distinct entry points produce a single downstream state. The receptor-access mechanisms, scaffold interactions and selected downstream signalling routes discussed in this section are summarised in Figure 2.

### 3.1. Location Bias Supplies the Specificity of the Trigger

Although functional selectivity contributes, a growing body of evidence assigns a major role to subcellular location bias [9]. Extracellular serotonergic signalling is constrained by limited access to intracellular compartments, whereas many psychedelics are membrane-permeable and can engage intracellular pools of 5-HT2A receptors, including those localised at the Golgi apparatus. Activation of this internal pool is sufficient to initiate structural neuroplasticity, including dendritic growth and spinogenesis, and artificially granting serotonin access to that compartment—for example, through the serotonin transporter (SERT), which restores its plasticity-promoting capacity. This is an elegant demonstration that the determining variable is location rather than ligand identity [9] (E1). Engagement of intracellular receptors thus provides a mechanistic route by which a transient exposure could initiate cascades that extend beyond acute signalling and ultimately couple to epigenomic programmes, including chromatin accessibility and enhancer activation [9,11].

Within this compartment, psychedelics accumulate in the Golgi through ion trapping and activate a receptor pool that mobilises local Ca^2+^ and engages transglutaminase 2 (TGM2) [9,46]; reviewed in [77]. TGM2 catalyses the serotonylation of the GTPase Ras-related C3 botulinum toxin substrate 1 (Rac1) at the glutamine 61 residue, a site otherwise central to Rac1 inactivation. Modification at this position impairs guanosine triphosphate (GTP) hydrolysis and renders Rac1 constitutively active [46]. This mechanism regulates actin polymerisation through p21-activated kinase 1 (PAK1) and is required for neurite growth and structural plasticity [46]. It is important to be precise about what has been demonstrated: receptor-stimulated transamidation of Rac1 at Q61 was established for plasma-membrane 5-HT2A/2C receptors in primary cortical cultures [46], and small G proteins are now recognised as the most common substrate class for monoaminylation [78]. The proposal that the Golgi-resident receptor pool is the compartment in which this reaction is preferentially triggered by psychedelics is an inference drawn here from the convergence of the two bodies of literature and remains to be tested directly (E3)—for instance, by compartment-restricted TGM2 inhibition.

### 3.2. The PSD-95 Receptosome Supplies Spatial Coherence

At the plasma membrane of a pyramidal neuron, activation of the 5-HT2A receptor by ligands including the psychedelic agonist DOI is not confined to a simple conformational change but involves assembly of a specialised multiprotein complex, or “receptosome” [10,79]. The core of this trigger resides in the intracellular C-terminus of the receptor, where a type I PSD-95/Dlg/ZO-1 (PDZ)-binding motif with a serine–cysteine–valine (-SCV) sequence acts as a molecular hook [47,79,80]. This site permits direct binding to PSD-95, which stabilises the receptor within the postsynaptic density, limits agonist-induced internalisation and sustains robust signalling [10,47,79]. PSD-95 is also required for the correct targeting and transport of the receptor toward apical dendrites [63,81], and disruption of scaffold interactions compromises dendritic receptor expression and downstream signalling [10]. The receptor additionally interacts with membrane-associated guanylate kinase (MAGUK) family members with inverted domain orientation (MAGI), MAGI-1/2/3, which act as enzymatic recruiters, facilitating the physical association of phospholipase C-β3 (PLCβ3) with the receptor and enhancing inositol phosphate formation; this particular functional effect is independent of the PDZ-binding motif itself [80].

Location bias thus supplies the specificity of the trigger and scaffolding supplies its spatial coherence. Neither, however, supplies duration, and it is to that problem that the remainder of this review is addressed.

### 3.3. Bifurcation into Cytoskeletal and Nuclear Routes

The canonical pathway proceeds through recruitment of the Gαq/11 protein [82], which activates PLCβ3 [82,83] and drives hydrolysis of phosphatidylinositol 4,5-bisphosphate (PIP2) into the second messengers diacylglycerol (DAG) and inositol trisphosphate (IP3) [80,84]. IP3 diffuses into the cytosol and binds the IP3 receptor on the endoplasmic reticulum, triggering Ca^2+^ efflux [12,82]. This calcium transient is the critical signal that engages protein kinase C (PKC) [80] and Ca^2+^/calmodulin-dependent protein kinase II (CaMKII) [12]. From this node the signal bifurcates along two strategic routes: one local and cytoskeletal, the other nuclear and transcriptional.

The rise in cytosolic calcium activates CaMKII [48], which targets Kalirin-7, itself anchored to the PSD-95 scaffold [48,49]. CaMKII phosphorylates Kalirin-7 on the Thr95 residue [48], converting it into a guanine nucleotide exchange factor for Rac1. This molecular switch activates p21-activated kinase (PAK) and promotes actin polymerisation [48,85,86]. The outcome is physical remodelling of the dendritic spine, producing a transient enlargement of the structure [46,48,49] and a strengthening of synaptic connectivity [48].

In parallel, the signal travels toward the nucleus through the mitogen-activated protein kinase (MAPK) cascade [8]. Both PKC and CaMKII converge on activation of the Raf/mitogen-activated protein kinase kinase (MEK)/extracellular signal-regulated kinase (ERK) 1/2 cascade [8,12]. Once phosphorylated, pERK translocates to the nucleus, where it cooperates with CaMKII to phosphorylate cyclic adenosine monophosphate (cAMP) response element-binding protein (CREB) on Ser133 [12]. CREB activation induces a psychedelic-associated transcriptional signature [12] comprising plasticity genes such as activity-regulated cytoskeleton-associated protein (*Arc*) and *Bdnf* (exons I and IV) [12], together with immediate early genes including *Fos* and early growth response 1 (*Egr1*) [82,87], whose regional induction patterns differ between psilocybin and ketamine [88]. Induction of *Egr2* has been proposed as the element that most reliably separates hallucinogenic from non-hallucinogenic 5-HT2A agonists [82,89,90], and thus as a candidate transcriptional correlate of the psychedelic response [87]; it should be noted, however, that this distinction was established in a limited set of ligands and brain regions, and that immediate early gene induction is dissociable from plasticity itself, since the non-hallucinogenic psychoplastogen tabernanthalog promotes neuroplasticity without proximate immediate early gene activation [1]. To preserve homeostasis, the system incorporates negative feedback: the ERK pathway activates ribosomal S6 kinase 2 (RSK2) [79], which returns to the membrane and phosphorylates the 5-HT2A receptor on Ser314 [8,63], attenuating signalling and preventing overstimulation.

### 3.4. Ligand-Dependent Divergences of Uncertain Weight

Two further branches are frequently invoked in this literature, and neither is treated as a required component of the enhancer-latching account.

First, while most agonists robustly engage the Gq pathway, selected psychedelic ligands such as 2,5-dimethoxy-4-methylamphetamine (DOM) and N-(2-hydroxybenzyl)-2,5-dimethoxy-4-cyanophenylethylamine (25CN-NBOH) have been reported to additionally recruit Gs/cAMP signalling in specific experimental systems [91]. In those systems, the rise in cyclic adenosine monophosphate mediated by adenylate cyclase behaves as a biochemical signature separating hallucinogenic from non-hallucinogenic agonists such as lisuride, and correlates with the head-twitch response [91]. This correlation should be interpreted with caution: it derives from a restricted ligand panel, and the head-twitch response is a rodent proxy for perceptual effects rather than a direct measure of them.

Second, recruitment of β-arrestin2 features prominently in accounts of biased agonism at this receptor [79]. Beyond its classical role in desensitisation and internalisation, β-arrestin2 also operates as a scaffold, assembling complexes including Src and protein kinase B (Akt) and initiating G-protein-independent cascades that influence dopaminergic neurotransmission [79]. Notably, in vivo work indicates that serotonin itself, but not *N*-methyltryptamines, activates 5-HT2A through this β-arrestin2/Src/Akt complex [92], which suggests that β-arrestin signalling may distinguish endogenous from psychedelic receptor engagement rather than mediating the latter. Its contribution to acute behavioural effects therefore remains debated.

### 3.5. TrkB as a Second Entry Point, and the Reconciliation of an Apparent Contradiction

In parallel with their action at G protein-coupled receptors, LSD and psilocin have been reported to act as positive allosteric modulators of TrkB, the receptor for BDNF, with affinities reported as up to three orders of magnitude higher than those of classical antidepressants [2]. This is a reported rather than a settled finding, and is treated as such from the outset because it is contested (see below). The transmembrane domain of TrkB contains a cholesterol-recognition (CARC) motif that confers cholesterol sensing and forms the antidepressant-binding site [93]. On the proposed mechanism, psychedelics bind a distinct but partially overlapping site within the transmembrane domain of TrkB dimers, stabilising the dimer and promoting its localisation to cholesterol-rich synaptic compartments where the receptor becomes more responsive to endogenous BDNF [2,93].

At this point the literature contains what looks like a flat contradiction, and a framework that builds on both sides of it is obliged to address the conflict rather than cite each side approvingly in different sections. On one side, in mice, the neurotrophic, plasticity-promoting and antidepressant-like effects of psychedelics require TrkB binding and endogenous BDNF signalling and were reported to be independent of 5-HT2A, whereas the head-twitch response requires 5-HT2A and is independent of TrkB [2,94]. On the other, conditional deletion of 5-HT2A in medial frontal cortex abolishes psilocybin’s effects on both stress-related behaviour and structural plasticity [7]. Taken at face value, the first result makes 5-HT2A dispensable for the durable component and the second makes it necessary.

The contradiction is partly apparent, because the two studies did not perform the same manipulation. Moliner and colleagues report direct binding to TrkB and plasticity that survives pharmacological antagonism of 5-HT2A; Shao and colleagues report a requirement established by targeted deletion of the receptor. Blocking the activation of a protein and removing the protein are different interventions, and a permissive or structural role for 5-HT2A—one not contingent on its agonist-driven activation—would render both results true simultaneously. That reconciliation is available, but it is a hypothesis to be tested rather than an established fact, and the experiment that would test it has not been conducted.

Recent work supports a functional convergence without settling the question [50]. Silencing TrkB abolished dendritogenic responses not only to serotonergic psychedelics but also to ketamine and to direct TrkB agonists, while silencing 5-HT2A selectively impaired psychedelic-induced plasticity and also attenuated responses to BDNF and to the TrkB agonist 7,8-dihydroxyflavone. Independent work in cellular systems reports that 5-HT2A and TrkB can occupy heteroreceptor complexes and that 5-HT2A availability modulates TrkB function [84], and PSD-95 postsynaptic localisation is itself regulated downstream of TrkB signalling [95]; both observations describe routes by which the two systems could be functionally coupled without an obligate physical complex. Three qualifications are required. The authors do not demonstrate an obligate physical complex between the two receptors and say so. The principal system is murine neural stem cells, which is not a single exposure in adult cortex, and the dependence varies with the output measured, so a statement of interdependence holds for some readouts and not automatically for others.

Four distinct propositions are at issue and are easily run together: that TrkB signalling contributes to the plasticity; that psychedelics bind TrkB directly; that the presence of 5-HT2A is necessary; and that the two receptors form an obligate physical complex. None entails the others. The direct-binding proposition in particular remains contested, since a broad polypharmacology screen of psychedelics reported no potent interaction with the kinases tested, TrkB among them [51]. We do not treat that screen as arbitrating the conflict on its own, because assay format and construct differ from those used to demonstrate a transmembrane allosteric site, but it must be discussed rather than omitted.

The evidence supports a functional convergence between 5-HT2A and BDNF–TrkB signalling that is dependent on system and on output (E2, contested). Independence from pharmacologically antagonisable 5-HT2A activation does not imply independence from the presence of the receptor. The contribution of direct psychedelic binding to TrkB, and whether an obligate physical complex is required, remain unresolved. The enhancer hypothesis developed below does not presuppose that these questions have been answered and is stated so as to survive either resolution. What does survive, independently of how the receptor controversy is resolved, is the dissociation of readouts—head-twitch from plasticity—which remains well supported and is what licenses the search for non-hallucinogenic psychoplastogens.

**Figure 3:**
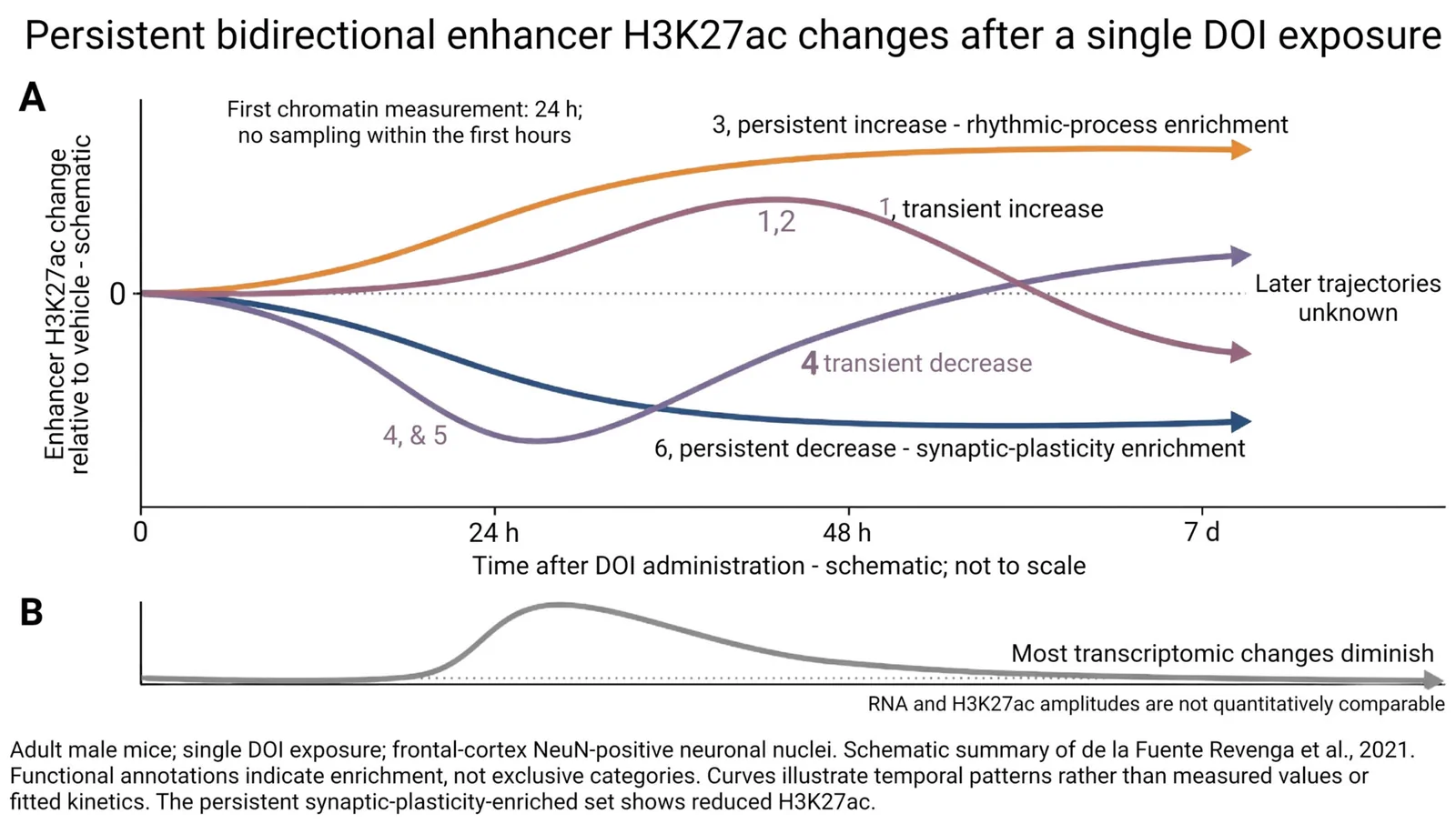
Persistent bidirectional enhancer H3K27ac changes after a single DOI exposure. (**A**) Schematic summary of H3K27ac profiling in frontal cortex NeuN-positive neuronal nuclei at 24 h, 48 h and 7 d after a single DOI dose in adult male mice [11]. Clusters 1 and 2 show transient increases peaking at 48 h, whereas clusters 4 and 5 show their deepest decreases at 24 h; these responses substantially diminish towards baseline by seven days. Cluster 3 remains elevated and is still rising at the final measurement, with enrichment for rhythmic processes. Cluster 6 remains depressed, with enrichment for synaptic-plasticity-related genes. Functional annotations are non-exclusive. (**B**) Qualitative representation of the predominantly transient transcriptomic response. Equivalence to baseline and locus-matched chromatin–transcript dissociation have not been established, and transcriptomic and H3K27ac amplitudes are not quantitatively comparable. Curves and temporal spacing illustrate temporal patterns rather than measured values or fitted kinetics. No chromatin measurements were obtained within the first hours or beyond seven days; subsequent trajectories remain unknown. DOI—2,5-dimethoxy-4-iodoamphetamine; H3K27ac—histone H3 lysine 27 acetylation; NeuN—neuronal nuclear antigen. The dose was 2 mg/kg intraperitoneally; animals were adult males; the time of day of tissue collection is not reported in the primary study. Created with BioRender, Cipriano, G. (2026) https://BioRender.com/39a8kax, accessed on 8 October 2026.

### 3.6. Convergence on PI3K/Akt/mTOR and GSK3β

These cascades converge on the phosphoinositide 3-kinase (PI3K)/Akt/mammalian target of the rapamycin (mTOR) pathway [5,82,92]. Akt promotes neuritogenesis, an effect linked to mTOR complex 1 activation in related contexts [89], and inhibits GSK3β at Ser9 [66], so pharmacologically distinct handles can produce a coherent downstream state. The dependence of this hub on PSD-95 requires a narrower statement: responses to DOI, including ERK1/2 phosphorylation, are impaired without PSD-95 in the system studied, alongside altered receptor expression [10]. That is not a demonstration that every entry into TrkB–PI3K/Akt/mTOR signalling is PSD-95-dependent; we therefore treat PSD-95 as a scaffold required where it has been tested, and the broader unification as a proposal.

Inhibition of GSK3β stabilises transcription factors such as CREB and orchestrates a coordinated programme of immediate early genes, including *Fos*, *Arc*, *Egr1/2* and *Nr4a1* [12,96], which in the PFC and limbic regions permits consolidation of neuronal growth after transient stimulation [97,98]. This is the first, still transcription-dependent layer of persistence; the layers that follow do not require sustained transcription, and their combination is what the latching model treats as the substrate of the consolidation window.

Two corrections are owed to the way this step is usually stated. The first concerns the nature of the inhibition. A serotonergic agonist is not a direct systemic inhibitor of GSK3β: the kinase is inhibited downstream of Akt activation, in whichever cells the receptor engages that cascade, so the effect is conditional on cell type and on signalling context rather than general to the tissue.

The second follows from it: reduced neuronal GSK3β activity need not impair consolidation. Inhibitory regulation of the kinase is integral to the relationship between long-term potentiation and long-term depression, and potentiation itself acts in part by inhibiting it [99]. That does not establish a universally favourable effect, but it invalidates the equivalence between lowered GSK3β and lowered neuronal plasticity, and with it much of the apparent paradox. What remains open is which cell types the drug modulates, over what duration and with what consequence for which programmes—quantities not measured after a single psychedelic dose (Section 5.2).

## 4. The Persistent Enhancer Landscape, the Set-Point Problem and Proteome Stability

### 4.1. What the Primary Data Show

The transition from an acute molecular trigger to stable plastic consolidation is a progression rather than a discrete event. Two architectural features are candidates for it: a persistent change in H3K27ac marking at enhancer elements [11] and stabilisation of the synaptic protein pool [14]. We name the measured quantity precisely, because the first is often described as a displacement of genomic accessibility. That is an interpretation, not the observation: what was profiled is an activating histone mark, and accessibility was not measured.

The chromatin evidence comes principally from a single study, and because the account examined here rests on it, its structure is set out in more detail than is usual in a review. A single dose of DOI in mice was followed by H3K27ac profiling of fluorescence-activated cell sorting (FACS)-purified, neuronal nuclear antigen (NeuN)-positive neuronal nuclei from frontal cortex at 24 h, 48 h and 7 days, with matched transcriptomics [11] (E1). There is no chromatin timepoint within the first hours; the earliest measurement is at 24 h, which matters for a claim examined below. Differentially marked enhancers resolved into six temporal clusters, and their shape bears directly on the relaxation account advanced in Section 2.3. Two features of that design bear on how the resulting structure should be read. The animals were adult males only, dosed at 2 mg/kg intraperitoneally, and the time of day at which tissue was collected is not reported, so any enrichment in rhythmic processes is compatible with a drug-induced phase shift as readily as with a change in mean level—a point developed in Section 4.3 but belonging here, where the measurement is introduced. The six clusters are a K-means partition in which the number of clusters was fixed by the analysts; they are a summary imposed on a continuum of temporal profiles rather than a structure recovered from the data. Since the bidirectional reading on which Section 4.2 depends is precisely the separation of a persistently elevated from a persistently depressed group, that reading inherits whatever arbitrariness the partition carries, and a reanalysis with cluster number selected by an explicit criterion is a prerequisite for treating the two-sided signature as a property of the data.

The scope of this dataset bounds every claim built on it. Its limits are collected in Section 8—one compound at one dose in one laboratory, adult male mice only, frontal cortex only, bulk neuronal nuclei rather than resolved cell types, an analyst-fixed cluster number, no chromatin timepoint inside the acute window and none beyond seven days, no reported cluster-level test against vehicle at that final timepoint, and no reported time of day for tissue collection—and are not restated at each point of use. Two consequences are stated here, where the measurement is introduced. Claims about persistence beyond seven days are claims about a trajectory that has not been measured, and are stated throughout as hypotheses: the multi-week evidence concerns the behavioural and structural phenotype, not the chromatin state. And because the measurement was made in animals of one sex, in one region and in one neuronal compartment of that region, neither the loci nor the directions reported here can be assumed to generalise across sex, region or neuronal subpopulation.

Four of the six clusters relax. Clusters 1 and 2 show increased acetylation peaking at 48 h, are enriched for axonogenesis, synapse organisation and cell junction assembly, carry Mef2B, NeuroG2 and Atoh1 motifs, and are reported as decreasing by seven days. Clusters 4 and 5 show decreased acetylation that is deepest at 24 h, are enriched for regulation of glutamate *N*-methyl-D-aspartate (NMDA) receptor activity, regulation of membrane potential and synapse assembly, and are reported to recover slowly over 48 h and seven days. Egr1 and Egr2 motifs are reported for clusters 5 and 6—that is, for one of the transiently depressed clusters and for the persistently depressed one.

Two clusters do not relax within the observation window, and their behaviour differs. Cluster 3 rises most slowly and is still rising at seven days, the last timepoint sampled; it is enriched for rhythmic processes and for the motif of NFIL3, a transcription factor of the circadian output pathway. Cluster 6 shows a decrease established by 24 h that remains at seven days, is enriched for synaptic-plasticity genes, and carries Egr1 and Egr2 motifs. Together these two clusters comprise at least 32.7% of all differential enhancers and remain in an altered state at seven days, against 3.7% of differentially expressed genes showing comparable long-term variation [11]. That cluster 3 has not been observed to relax at all is a material limitation on the account in Section 2.3, and we return to it there.

The cluster labels should not be read as exclusive categories. Terms relating to synapse organisation and assembly are enriched, to differing degrees, across the clusters rather than partitioning cleanly between a “circadian” set and a “synaptic” set. What distinguishes clusters 3 and 6 is the direction and the persistence of the change, not a disjoint functional annotation, and any interpretation that relies on the labels being mutually exclusive is unsupported. The principal enhancer trajectories and their contrast with the predominantly transient transcriptomic response are summarised schematically in Figure 3.

Three observations follow, and the first two are, to our knowledge, not previously drawn.

The four relaxing clusters are consistent in shape with what Section 2.3 requires, although without a seven-day test against vehicle their return is described rather than quantified. A model claiming slow relaxation rather than a fixed displacement expects measurable decay between 48 h and seven days across much of the affected landscape, with a slow residual. Three qualifications attach to this reading: it is retrodictive; the sampling is too sparse to estimate any time constant; and the persistently elevated cluster supplies no relaxation to fit, since it has not yet turned over at the last timepoint.

The persistently elevated set carries a circadian signature that the persistently depressed set does not. This is the feature of the dataset most consequential for the enhancer-latching account: the persistent elevated set is routinely characterised loosely as the plasticity-associated one. The plasticity-annotated persistent set is the depressed one. Section 4.2 addresses what this means for the directional reading, and Section 4.3 examines whether it points toward a maintenance mechanism.

We report the 32.7% and 3.7% figures because they are the published summary of the dataset, but we decline to treat their ratio as a quantitative result. Chromatin immunoprecipitation sequencing and RNA sequencing differ in detection sensitivity, in multiple-testing burden and in the statistical structure of their null, and the two percentages have different denominators; the ratio is therefore not a biologically interpretable quantity, and a matched-power reanalysis of the deposited data would be required before any such comparison could carry argumentative weight.

A further clarification is owed regarding the locus-level claim, and it is more damaging than it first appears. The strongest form of the argument would be a locus-matched dissociation: the same gene showing a persistently altered regulatory element and an unaltered transcript at the same timepoint in the same animals. Such a comparison is often taken to be immune to differences in assay sensitivity between platforms. It is not. A transcript that is not significantly different from control is not thereby equivalent to baseline, and a locus-matched design reduces but does not remove the asymmetry in statistical power and detection floor between chromatin immunoprecipitation sequencing and RNA sequencing. Establishing the dissociation requires an equivalence test against a pre-specified margin [100], not a failure to reject the null.

Per-gene directional data at seven days for *Bdnf*, *Dlg4* and *Arc* are in any case not reported in the primary publication, so we do not assert the locus-matched dissociation as established. What is established is a cluster-level dissociation: enhancer sets annotated to synaptic function remain displaced at seven days while the transcriptome has largely normalised, with the caveat about equivalence just stated. Extracting per-locus direction from the deposited data and testing it as an equivalence rather than as a null result, is the first item of the reanalysis programme this review implies.

One further point of evidential status must be recorded. The seven-day DOI signature is not the only psychedelic epigenomic dataset in the literature: H3K27ac has since been profiled after psilocybin in frontal cortex and nucleus accumbens, with and without prior oxycodone exposure, and the enhancer-level changes reported there are dependent on sex and on region [52]. This is additional evidence, not an independent replication of the DOI cluster structure at seven days, and it does not relieve the single-dataset vulnerability described in Section 8. The point must be put sharply. In that study tissue was collected on the day after psilocybin administration, so the dataset characterises the acute epigenomic response and supplies no information at all about the seven-day persistence that the enhancer-latching account is about.

### 4.2. Bidirectionality and the Directional Assignment

The persistent fraction comprises two clusters of opposite signs, and any model that describes psychedelic action purely as a gain of enhancer activity is incomplete (Figure 3A). Latching is therefore a bidirectional and structured displacement of the enhancer landscape.

This bidirectionality is the principal internal tension in the account, and it is easily resolved too quickly in its favour. A persistent decrease at enhancers of synaptic plasticity genes admits at least three readings, and the data do not distinguish them. It may reflect a durable reduction in programmes that constrain remodelling. It may arise as homeostatic compensation following acute activation. Or it may reflect a reduction in capacities that the consolidation of plasticity requires, in which case its sign is opposed to a permissive reading.

One clarification is needed about the second of these, because it is easily dismissed by calling it a consequence of the drug response rather than a substrate. That does not follow. The origin of a signature does not determine its causal role: a change that arises as compensation for an acute event can perfectly well go on to set thresholds for a later one, which is precisely the kind of mechanism this account proposes. A persistent homeostatic adjustment could therefore mediate subsequent metaplasticity, or accompany it without contributing—and those two possibilities are separable by timed perturbation, whereas the question of where the signature came from is not the question of what it now does. The alternatives must be framed to separate the origin of the signature from its subsequent function rather than conflating them.

The motif enrichment supplies a more specific handle than the ontology terms do, but it does not by itself favour any of the three readings. The Egr1 and Egr2 motif enrichment described in Section 4.1 is the relevant observation here. It is tempting to argue that early growth response (Egr) programmes consolidate and thereby close plasticity down, so that their persistent decommissioning removes a brake. That inference does not follow: consolidating a change and preventing future change are different functions, and the evidence runs the other way at least as strongly, since gain of Zif268/Egr1 function facilitates hippocampal synaptic plasticity and long-term memory rather than restricting them [101]. Reduced output from Egr-motif elements is therefore at least as compatible with diminished plastic capacity as with released constraint.

Three further limits on what motif enrichment can support should be stated, because they apply to every directional argument in this section. Enrichment of a motif does not demonstrate occupancy of the corresponding factor; occupancy does not demonstrate activity; and neither identifies which genes the elements actually control, since enhancer–gene assignment by proximity is unreliable [102]. Nor is H3K27ac a direct measure of accessibility or of activation threshold [54]. A lowered threshold is a claim about re-inducibility and must be measured as such; it cannot be read off the sign of a histone mark. What remains worth testing, and is cheap, is whether the acutely Egr2-responsive elements are the same elements that persist in cluster 6—but the dataset has no chromatin timepoint inside the acute window, so this cannot be answered from the existing data. A narrative of acute engagement followed by durable decommissioning is therefore not available, however natural it looks.

The directional interpretation is a hypothesis and not a finding (E3), and its status is auxiliary in the sense of Section 2.4. It requires that the persistent gene sets be classified functionally into permissive and constraining categories, with the classification fixed before the direction is examined. Standard ontologies will not do the work: elements annotated to synapse assembly appear on both sides of the signature. The falsifying outcome must also be scoped correctly. If the constraining set retains or gains activity while the permissive set loses it, what is excluded is this directional interpretation of the signature, not every version of an enhancer-based account. A configuration could contribute causally to altered capacity while the sign of its elements maps onto function in some way we have not anticipated, or in no simple way at all.

A limitation of cellular resolution qualifies all of the above. These data were obtained from the entire NeuN-positive neuronal population rather than from resolved cell types [11], so the possibility that opposing changes in excitatory and inhibitory neurons are being averaged together cannot presently be excluded. This is not a minor caveat: it means the bidirectionality reported at the population level may reflect cell-type heterogeneity rather than functional heterogeneity within a cell type, and the two possibilities make different tests under Test 3. It is also the same caveat that Section 2.1 raised against dismissing sustained sparse transcription, and it applies with equal force in both directions.

### 4.3. What Could Conserve the Difference

The mechanistic objection stated in Section 2.1 has to be answered here. Because no acetyl group deposited at the time of exposure is present seven days later, persistence of the mark is not persistence of a molecule but persistence of a set-point: a durable change in the equilibrium between p300/CBP-mediated deposition, HDAC-mediated removal and local acetyl-CoA availability (Figure 1B).

Framed this way, the question becomes what holds the equilibrium displaced. One answer is that only an anchor extrinsic to the cell, or one driven by a recurrent external input, could terminate the explanatory regress. There is no such necessity. A slowly reversible intracellular state variable, or a feedback loop sustained by ordinary metabolism, can conserve a difference without requiring an infinite succession of maintainers. Nor does an extracellular position confer memory: the extracellular matrix does not terminate a regress, because one must still explain how it is modified, why it retains the modification, and how it communicates that modification to the nucleus. Relocating the boundary of the system is not the same as identifying the mechanism.

We therefore replace the criterion. An adequate maintainer must satisfy three requirements: it must be an identifiable physical variable rather than a placeholder; its dynamics must be slow enough to span the interval in question; and its coupling to the enhancer configuration must be verifiable rather than assumed. On these requirements, five candidates are worth examining, and none of the five currently qualifies (all E3; Table 2).

The first is nucleosome turnover. Histone exchange in post-replicative cells was long assumed static, but the variant H3.3 accumulates to near-saturating levels across the neuronal genome by mid-adolescence while remaining highly dynamic in a modification-independent manner, and histone cell cycle regulator (HIRA)-dependent histone turnover regulates activity-dependent transcription, synaptic connectivity and behaviour [56]. The candidate variable would be the rate of exchange rather than the occupancy. It is best understood as a possible proximate implementation of a displaced set-point rather than as an explanation of what sustains one.

The second is local acetyl-CoA availability. This term appears in the equilibrium above but is rarely pursued, although it is the most direct route by which cellular state sets acetylation capacity. Nuclear acetyl-CoA production is not necessarily uniform, since acetyl-CoA synthetase 2 associates with chromatin at neuronal genes and couples local metabolite supply to local histone acetylation and to memory [36,58]. This makes the candidate more interesting rather than less, because a locally targeted metabolic supply could in principle carry pattern as well as duration—but it also removes the argument that a metabolic anchor must act globally.

The third is the state of the viscoelastic extracellular matrix, which is remodelled within 24–48 h and which has been shown to feed back onto nuclear architecture and cis-regulatory accessibility [55]. Two qualifications are needed. That work is conducted principally in fibroblasts and in the context of cellular reprogramming, not in adult cortical neurons after psychedelic exposure; it is a precedent for plausibility, not a demonstration of the bridge invoked here. And, as noted above, the matrix’s extracellular position does not by itself supply memory.

The fourth is circadian gating, and it requires the most correction. The persistently elevated enhancer set is enriched for rhythmic processes and for the NFIL3 motif (Section 4.1), and recurrent circadian re-entrainment is the natural candidate for the input that holds the displaced set-point. That proposal has a defect that is easily overlooked. Treated and control animals normally receive the same light–dark cycle. If the same input re-establishes two different configurations, then a difference in the response to that input must already be stored somewhere—in oscillator phase, in coupling strength, in factor occupancy, in chromatin architecture, or in some other variable. That difference is precisely the substrate still to be explained. A circadian cycle can time the maintenance of a state; it cannot by itself supply the address and the sign of what is maintained.

The motif evidence is also weaker than it first appears. Motif enrichment does not demonstrate NFIL3 occupancy, and NFIL3 recognises specific sequences and can act as a repressor competing with activating proline- and acidic-amino-acid-rich (PAR) family factors at the same sites [61]; its motif therefore does not identify an undifferentiated genomic power supply. A further confound must be excluded before any circadian test is interpretable: H3K27ac itself oscillates over the twenty-four-hour cycle at enhancer elements, in the suprachiasmatic nucleus and in liver [57,59], so a difference measured at a single clock time may reflect a phase shift rather than a change in mean level. Demonstrating that in those tissues does not demonstrate it in prefrontal cortex, but it makes controlling for it obligatory. Any circadian experiment must separate change in mean, change in phase and change in amplitude, and existing data do not.

A fifth candidate deserves separate treatment, because it is the only chromatin modification directly coupled to the transmitter system under discussion and because the same enzyme has already been invoked at the trigger. Transglutaminase 2 transfers serotonin to glutamine 5 of histone H3; H3Q5ser is combinatorially coupled to H3K4me3, enhances TFIID binding and marks active regulatory regions, and its levels track serotonergic state [29]. The analogous dopaminylation mark in ventral tegmental area is dynamically regulated across weeks of withdrawal and causally controls drug seeking, which establishes that a monoaminylation mark can hold a behaviourally relevant state over the interval at issue here [30,60]. We nonetheless regard it as the candidate to prioritise, for two reasons that are specific rather than general. The enzymatic machinery is the transglutaminase 2 already invoked at the intracellular trigger in Section 3.1, so a single manipulation addresses both ends of the account. And unlike a circadian input, a mark deposited by the serotonergic system at specific sequence contexts could in principle supply address and sign together, which is exactly the property argued above that the other candidates lack. This is a reason to measure it, not evidence that it is the maintainer, and the account does not depend on the outcome.

Any account of this kind is obliged to name a recurring input carrying the relevant specificity, and circadian re-entrainment does not carry it, for the reason given above. What the evidence supports is that any such account requires a state variable or feedback dynamic capable of conserving an exposure-induced difference despite acetylation turnover, that extracellular matrix, metabolism and circadian oscillators are candidates for modulating its maintenance, and that their extrinsic or periodic character neither establishes them as the maintainer nor excludes them from contributing to the information conserved.

These five are candidates rather than a conjunction, and none is currently established. The requirement that cannot be relinquished is that some variable meeting the three conditions exists and couples to the enhancer configuration; in the absence of such a variable the account has no mechanism. Excluding these five would not, however, exclude the concept of a conserved regulatory configuration, which could be sustained by a mechanism not yet proposed.

One further objection is the sharpest available against any such account. If the displaced set-point is held by some maintainer, then the maintainer is the store, the enhancer landscape is its downstream readout, and the substrate has been located in the wrong place. A distinction between information and maintenance is the reply available in the literature; it is set out here together with the reasons why the available evidence does not establish it. The proposal is that a maintainer supplies durability while the enhancer configuration supplies specificity—which loci, which sign, and therefore which subsequent stimuli are answered differently—so that the store is the pattern and the maintainer is what keeps the pattern alive, in the sense in which the contents of a volatile memory are held in the memory rather than in the supply that powers it.

The argument usually available for this rests on the claim that every candidate maintainer is locus nonspecific. That claim is not secure. Chromatin-associated acetyl-CoA synthetase couples metabolite supply to acetylation at particular neuronal genes [36], and NFIL3 binds specific sequences rather than acting as an undifferentiated input [61]; a perturbation of maintenance can therefore have locus-specific consequences. The distinction between information and maintenance remains conceptually available and is the most precise way to state what is at issue, but it is at present a proposal rather than a result.

It also cannot be settled by a rate-versus-composition test. The natural proposal is that manipulating a maintainer should change the rate at which the persistent sets relax while leaving their composition unchanged, so that a change in composition would locate the information in the maintainer. That inference fails twice over. A common perturbation can accelerate decay differentially across loci that differ in sensitivity, producing a compositional change with no relocation of information whatever. And because set membership is defined by a significance threshold, any manipulation that reduces amplitude will push marginal elements out of the significant set, so composition will appear to change for purely statistical reasons. Rate and composition are not independent quantities under a thresholded readout, and the contrast between them does not localise a store.

What would be informative is harder and more specific. To support a causal role for the configuration one would need a local normalisation of the state that selectively abolishes the acquired responsiveness while the maintenance inputs remain operative, followed by targeted restoration of the state and recovery of the responsiveness.

That design is a genuine advance on global deacetylation, but it should not be claimed to deliver more than it does. Selective normalisation plus rescue supports a causal role for the configuration without uniquely identifying the primary store. The result is equally compatible with an arrangement in which the persistent state is conserved elsewhere, the enhancer configuration is a necessary downstream element, and the modified response requires it: normalising the enhancer would abolish the response and restoring it would recover the response under that arrangement too. What has been demonstrated is selective causal mediation, which is not the same as localisation of information.

Approaching localisation requires following the fate of the state after the editor is withdrawn. Does the configuration reconstitute spontaneously, which would indicate that the instruction resides upstream? Does the erasure persist, which would indicate that the configuration is not being continuously specified from elsewhere? Does rewriting the pattern de novo, in an animal that never received the drug, confer responsiveness? These outcomes constrain different models without any single one being decisive, and we regard assembling them as the realistic path to deciding whether the configuration is the store or a necessary mediator—rather than a single experiment that settles it.

Because the turnover premise is imported from other systems rather than measured at these elements (Section 2.1), the consequence of its failure should be stated. If acetyl turnover at the persistent elements proved slow—if they fall among the minority of sites with substantially slower kinetics, if histone exchange at these loci is reduced, or if the local balance between deposition and removal is shifted toward retention—then the set-point reformulation is unnecessary and the simpler account becomes available: the mark itself is retained, and the maintenance problem set out in this section dissolves into a question about turnover at specific loci. The experimental programme changes accordingly, from identifying a maintainer to measuring exchange. A third possibility is neither, and the available data cannot exclude it: a change in cellular-state composition within the NeuN-positive population rather than a change in marks, in which a stable subpopulation rather than a stable modification carries the persistent signature. Bulk profiling cannot distinguish that from a uniform shift; single-nucleus profiling would.

### 4.4. Post-Transcriptional and Proteomic Stabilisation as a Competing Account

To bridge these marks and longer-term outcomes, persistent cell-type-specific alternative splicing changes have been reported in the prefrontal cortex following a single dose, lasting at least one month, with functional enrichment connecting to the ECM and to synaptic and intrinsic physiology in parvalbumin interneurons over a shorter window of days to a week [62]. This work remains a preprint with no peer-reviewed version at the time of writing. It is cited as consistent with the account rather than as support for it, and no test in Section 2.5 depends on it. A replication adequate to change that status would require cell-type resolution, at least two structurally distinct compounds, and at least two timepoints beyond seven days.

The maintenance of synaptic function requires a second regulatory level: protein stability [10]. Synaptic vesicle protein 2A density measured by autoradiography remains elevated at seven days after a single dose of psilocybin in pig [14]. That study quantified protein-level densities and did not measure transcript levels, so the dissociation between persistent synaptic markers and a normalised transcriptome must be assembled across studies and systems rather than read from that one. The same principle of decoupling is separately supported by the observation that, in the absence of PSD-95, messenger RNA levels of serotonergic receptors remain stable while functional protein expression falls markedly [10]. Maintenance of PSD-95 levels is in turn linked to synaptic resilience: transcriptional control of PSD-95 expression influences synaptic density in ageing and in neurodegenerative contexts [103].

Enhancer displacement, scaffold stabilisation and post-transcriptional control are easily presented as three nested timescales rather than three competing explanations. That framing is too convenient: if a long-lived, palmitoylation-locked scaffold pool is sufficient to alter how a synapse responds to subsequent stimulation, proteostatic persistence is a complete alternative substrate and does not require a chromatin store. We therefore treat it as a competing account (E1 for the individual components, E2 as a substrate of psychedelic persistence), and the observation that would separate it from ours is given in Section 7.

The discriminating experiment must be stated carefully. It might seem that if blocking PSD-95 palmitoylation shortens the persistence window while the enhancer landscape remains displaced, the proteostatic account supersedes this one [25]. That does not follow. PSD-95 may be required to express a capacity that is conserved at the chromatin level, and interrupting execution does not identify an alternative store any more than interrupting the enhancer identifies a chromatin one. Loss of the phenotype after scaffold perturbation, with the enhancer configuration intact, demonstrates a scaffold requirement; it leaves an informational chromatin component entirely possible.

### 4.5. Routing, Structure and the Wider Environment

Consolidation of this physical rewiring allows structural plasticity to be decoupled from acute perceptual effects, as shown by non-hallucinogenic analogues that promote spinogenesis without eliciting the head-twitch response [1,104]. The routing evidence must be stated carefully. Psilocybin increases spine density in both pyramidal tract and intratelencephalic populations, while silencing pyramidal tract neurons, but not intratelencephalic neurons, eliminates the behavioural outcomes examined [7]. That establishes a circuit requirement for expressing those particular effects. It does not establish that every persistent molecular trace is confined to that class, and the natural inference—that persistence is therefore not a diffuse cortical property—conflates the circuit needed to express an effect with the distribution of its possible substrate. What the result does establish is a resolution problem: the chromatin measurements were made across the whole NeuN-positive population, which contains both classes and much else besides, so the signature cannot presently be assigned to the population that carries the behaviour.

Beyond the neuron, specific psychoplastogens such as ibogaine increase the expression of cerebellins (*Cbln2*, *Cbln4*), which act as trans-synaptic organisers physically stabilising newly formed synaptic contacts [105,106]. Such remodelling occurs alongside regulation of ECM-associated genes, whose reorganisation is fundamental to the reopening of critical periods of plasticity [41,42,43,72]. Recent findings indicate that the viscoelastic properties of the ECM can enhance nuclear remodelling and epigenetic accessibility at cis-regulatory elements associated with neuronal genes [55], suggesting a mechanical route by which the extracellular environment might feed back onto chromatin state. Its extension to mature cortical neurons after psychedelic exposure remains an extrapolation (E2; Section 4.3). This biological flexibility supports a reconfiguration of neural dynamics characterised by a relaxation of high-level priors and reduced neuronal correlations in the orbitofrontal cortex [107]; that dataset is likewise a preprint. At the psychological level, a preliminary within-subject study in a small non-clinical sample found that confidence in negative self-beliefs decreased after 25 mg psilocybin and that this decrease was associated with increases in well-being at four weeks [108]; the sample is small, non-clinical and the follow-up short, so this evidence is suggestive of a mediating psychological process rather than demonstrative of one.

## 5. The Glial Permissive Branch

For a persistent regulatory configuration to be established and expressed, the neural microenvironment must be sufficiently permissive [2,17]. In neurodegeneration and brain ageing, chronic neuroinflammation acts as a brake on plasticity and accelerates neuronal atrophy through microglial hyperactivation; an account of durable plasticity that ignored this constraint would predict persistence in precisely the tissue least able to sustain it.

Two propositions must be separated. That a sufficiently permissive environment is required for durable remodelling is a general statement about tissue and is not in dispute. That drug-induced glial modulation is necessary for the enhancer trace—particularly in uninflamed tissue, where there is little constraint to lift—is far stronger and untested (E2/E3), and we develop it as an auxiliary hypothesis in the sense of Section 2.4, most pertinent in inflammatory contexts and not assumed by the causal core.

We ask whether psychedelics act on this constraint through a coordinated mechanism we term neuro-immune gatekeeping [4,109], assembled from separately established links rather than demonstrated as a causal chain. The claim the section defends is that neuro-immune permissiveness is a plausible enabling condition for persistence, tested by Test 5 whichever molecular route implements it, and not that it is a demonstrated requirement.

### 5.1. Compartmentalising NF-κB

NF-κB cannot simply be cast as a brake on plasticity: neuronal NF-κB is required for structural plasticity and long-term memory, and ablation of its subunits impairs axon outgrowth, synaptogenesis and hippocampal tissue homeostasis [110]. Genetic models support roles that differ by cell type, inhibition in glia being protective in disease contexts and activation in neurons supporting memory [111]. The gate at issue is glial rather than neuronal.

Two qualifications keep this from being a clean dichotomy. Chronic activation of neuronal NF-κB can itself recruit repressive programmes through histone deacetylase 2 (HDAC2) [69], a negative regulator of memory and synaptic plasticity [70], so initial state, duration and target set matter as much as cell identity. And the usual measurement may miss the relevant effect: in lipopolysaccharide-stimulated microglia, inactivation of GSK3β reduced p65 transactivation and tumour necrosis factor α production without reducing p65 nuclear translocation or DNA binding [68], so assaying translocation alone could return a null.

The prediction must also be weakened: glia-restricted NF-κB inhibition removes a constraint but does not supply an instruction, since lifting a brake may permit a trace to be written without specifying what is written. The informative design is factorial—drug versus vehicle crossed with glial manipulation—and must distinguish inflamed from basal tissue, where a permissive manipulation has little room to act.

### 5.2. What Has Not Been Measured

What is missing at this compartmental resolution is not a paradox but data: cell-type-resolved measurement of GSK3β activity and of NF-κB transactivation—not merely translocation—after a single psychedelic dose, at the timepoints over which the enhancer signature develops. To our knowledge this has not been reported for any psychedelic, and until it is, statements about which compartment the drug acts in are inferences from lipophilicity rather than observations.

### 5.3. Why the Glial and Neuronal Branches Are Parallel, Not Serial

A sequence has been proposed in this literature, running from inhibition of GSK3β to reduced nuclear translocation of glial NF-κB, thence to reduced transcription of the negative epigenetic regulator HDAC2 and thence to a displaced acetylation set-point at neuronal enhancers [66,67]. It cannot be stated as a serial chain, because it changes cell type midway without a mediator.

The NF-κB-to-HDAC2 link is characterised for chronic antipsychotic treatment in forebrain cortical pyramidal neurons, where deletion of HDAC2 in those neurons prevents the synaptic and cognitive consequences of the drug [69]: it is neither a glial mechanism nor a psychedelic one, and the two remaining steps are drawn from the antidepressant [66] and antipsychotic [67] literatures, so no step has been established after a psychedelic. Either HDAC2 changes in glia, in which case the signal reaching the neuron is unidentified—glial HDAC2 does not deacetylate neuronal chromatin—or it changes in the neuron, in which case the link from a glial perturbation to its neuronal control is missing. Neither version has been demonstrated.

We therefore separate the two branches and the claims made about them: the glial branch modulates the permissive environment, the neuronal branch regulates enhancers, and they are currently parallel rather than serial. The serial chain can be reintroduced only by specifying and demonstrating the intercellular mediator—a cytokine, a growth factor, a change in ambient signalling—that carries the glial state into the neuronal nucleus. Without it this route is not a continuous mechanistic explanation; the enhancer hypothesis is untouched, because it does not rest on it.

### 5.4. Better-Characterised Glial Actions

Downstream of this putative gate, direct immunomodulatory actions on glial cells are better characterised and constitute the strongest evidence in this section [71]. Psilocybin and psilocin suppress tumour necrosis factor α expression and increase BDNF in lipopolysaccharide-activated microglia, with aryl hydrocarbon receptor (AhR) signalling required for psilocin-induced BDNF upregulation but not for cytokine suppression [112]. *N*,*N*-dimethyltryptamine (DMT) restores tight junction integrity and blood–brain barrier function and reduces microglial activation through the Sigma-1 receptor, a chaperone enriched at the endoplasmic reticulum–mitochondria interface [113,114,115]; Sigma-1 modulation by DMT has also been examined in an amyloid-β model [116]. The immunomodulatory capacity of these compounds has been reviewed more broadly elsewhere [117].

Three connections between this branch and the rest of the account are best put as measurable questions rather than as a chain. First, whether an anti-inflammatory window is required for the persistent alternative splicing reported in prefrontal cortex [62] is testable by crossing a glia-restricted manipulation with drug exposure and reading splicing at one month. Second, whether Sigma-1-dependent support of mitochondrial homeostasis alters nuclear acetyl-CoA availability is directly measurable, and is the only point at which this section touches the maintenance problem of Section 4.3. Third, whether inflammatory state alters the viscoelastic properties of the perineuronal matrix in adult cortex, as opposed to the non-neuronal systems in which that coupling was characterised [55], is unmeasured.

## 6. Therapeutic Versus Pathological Latching: The Missing Selector

The capacity to modulate plasticity through chromatin-level mechanisms is not without risk. In contexts of repeated or high-dose exposure, substances such as MDMA and ketamine have been associated with maladaptive epigenetic remodelling, including altered DNA methylation and reduced histone acetylation [17]. The same machinery that supports therapeutic latching can, under different exposure regimes, latch a pathological state. A framework that cannot distinguish the two at the molecular level has limited translational value, and we therefore specify a criterion rather than leaving the distinction to outcome.

The obvious discriminant is response direction: if a psychedelic lowers the cost of re-entering the plastic state, therapeutic latching should predict amplified re-induction and pathological latching occlusion, with plasticity spent rather than primed. A counterexample defeats that ordering. The best-characterised pathological latch in this literature is behavioural sensitisation to psychostimulants, and sensitisation produces amplified, not occluded, responses to a subsequent challenge [76]. As a primary criterion, amplification versus occlusion maps backwards onto the paradigm case.

Locus identity is the natural second attempt: therapeutic latching would involve synaptic-assembly and BDNF-TrkB loci, pathological latching stress, immune and reward-sensitisation loci. That proposal does not survive either, and the reason is instructive. A cell does not distinguish therapeutic from pathological as biological categories. The loci we assigned to the therapeutic category are not therapeutic in themselves: BDNF–TrkB signalling in the nucleus accumbens contributes to cocaine reward, with effects that differ by neuron type [73], and targeted epigenetic remodelling at the *Fosb* locus alters responses to cocaine and to stress [74].

It follows that identical locus sets across the two regimes would not exclude a shared metaplastic substrate. They would exclude only an exclusive locus-valence classifier, which is therefore not part of this account. The translational claim must be correspondingly weaker. It does not postulate intrinsically therapeutic or pathological elements; cis targeting depends on the interaction of pre-existing accessibility, activity-recruited factors, cofactors and enhancer–promoter contacts, and the configuration of these after psychedelic exposure remains to be identified. The unit of comparison is the regulatory state of a specified element in a specified population—direction, amplitude and duration together—whose adaptive valence depends on circuit and experience.

This leaves the account owing a selector, and none is available (E3). A combinatorial selector is at least plausible: depolarisation, BDNF and forskolin can induce *c-fos* through different combinations of enhancers and factors, so pre-existing accessibility, activity-recruited factors and cofactor availability can in principle specify which elements a given stimulus engages [75]. That establishes that such mechanisms exist; it does not identify the psychedelic one. A methodological hazard comes with this admission and should be stated: if every discordant result can be attributed after the fact to a different context or cofactor, no result can decide the question. The context variables must therefore be specified and bounded before analysis rather than invoked afterwards.

The authors report that differential H3K27ac peaks overlap genomic loci associated with schizophrenia, major depressive disorder and attention deficit hyperactivity disorder, and present this as a caution attaching to their own finding rather than as a therapeutic result [11]. The overlap is reported for static peaks as well, so it is a property of the cortical enhancer landscape rather than of the elements the drug selects, and it identifies no selector. What it does illustrate is that any intervention on that landscape operates in a genomic neighbourhood dense with psychiatric risk, which raises the evidential bar for any future proposal claiming to separate the therapeutic from the pathological regime by which loci are involved.

The dosing prediction requires a narrower statement. From a slow relaxation constant, it follows that residual states from successive exposures overlap when the interval is shorter than the relaxation time. It does not follow that they saturate, occlude or become pathological: a slow integrator can accumulate without occluding, and the conversion requires an additional nonlinearity that has not been demonstrated and that the account does not specify. What the account implies is overlap of residual states; anything beyond that is a further hypothesis requiring its own justification.

Occlusion, if sought experimentally, must also be distinguished from three things it resembles. A ceiling effect in the readout, receptor-level tolerance, and reduced activation upstream of the nucleus all produce a smaller response to a second dose, and none implicates a chromatin memory. A diminished second response is not by itself evidence for anything this account proposes.

## 7. Discriminating Enhancer Latching from Competing Frameworks

The field now contains several unifying accounts of psychedelic action—metaplasticity-based, critical-period and ECM-based, methylation-based, excitation/inhibition-based and learning-based—and comparatively few experiments capable of separating them. Because these accounts are largely compatible at the level of phenomenology, the practical contribution of a substrate hypothesis is to specify where they come apart. Table 3 states, for each of the principal alternatives, an observation that would favour one account over the other.

The table includes three accounts that are easily absorbed as supporting layers rather than stated as rivals: proteostatic persistence, sustained sparse transcription, and the null account on which the seven-to-thirty-day window requires no dedicated molecular store beyond ordinary consolidation. The last of these deserves particular emphasis, because it is the most parsimonious alternative available and because the scope restriction in Section 2.2 concedes its logic for the multi-year regime. If ordinary consolidation is what maintains benefit after the first month, the burden falls on any chromatin-based account to say why it is not also what maintains it during the first month, with the enhancer signature a correlate of the consolidating circuit rather than its store.

Three features of this table deserve comment. First, several rows require reciprocal tests rather than single experiments, because a manipulation that abolishes persistence is compatible with more than one substrate; only the pairing of forward and reverse manipulations is diagnostic. Second, none of these experiments has been performed. Third, the matrix is not exhaustive: it lists the accounts current in the field at the time of the search stated in the section Scope and Literature Selection, so exclusion of every alternative listed here would not establish enhancer latching by elimination, and a substrate not yet articulated remains available.

**Table 3:** A substrate discrimination matrix. Observations that separate the enhancer-latching account (EL) from the other accounts of psychedelic persistence current in the field. Each row states a result that would favour the competing account over EL, or the reverse. The evidence level of each competing account’s own substrate claim (E1–E3, defined in the note to Table 1) is stated in the second column. None of the discriminating observations listed has been reported. Abbreviations: 5hmC—5-hydroxymethylcytosine; 5mC—5-methylcytosine; E/I—excitation/inhibition; ECM—extracellular matrix; H3K27ac—histone H3 lysine 27 acetylation; H3Q5ser—histone H3 glutamine 5 serotonylation; PSD-95—postsynaptic density protein 95; PV—parvalbumin; TGM2—transglutaminase 2.

Competing Account	Proposed Substrate	Discriminating Observation	Refs.
Metaplastogens	E1 for the phenomenon; no substrate claim. Substrate not specified	EL requires persistent enhancer displacement. Durable metaplasticity demonstrated with an enhancer landscape shown equivalent to naive within a pre-specified margin would exclude EL while leaving the metaplasticity account intact	[40,100]
Critical periods/ECM remodelling	E1 for critical-period reopening; E2 for the matrix-to-chromatin link in cortex. ECM and perineuronal net state	Manipulate the ECM after the enhancer window has closed. Persistence that survives would exclude chromatin as the store; persistence that is abolished is compatible with either, and requires the reciprocal test (enhancer manipulation with ECM intact)	[41,42,43,72]
Structural trace (drug-induced spines)	E1, but demonstrated for antidepressant-induced spines rather than after a psychedelic. Newly formed dendritic spines as the store	Selectively ablate the spines formed after the drug while leaving the enhancer landscape displaced. Loss of the maintained effect with displacement intact favours the structural trace; persistence of the effect after ablation favours EL. The reciprocal test—normalising the enhancer landscape with the new spines intact—is required before either result is read as decisive	[26]
DNA methylation-based accounts	E2; rapid experience-driven changes are established, but methylation has not been profiled after a psychedelic at matched timepoints. 5mC/5hmC at cis-regulatory elements	Paired whole-genome bisulfite and H3K27ac profiling at matched timepoints. If persistence tracks methylation and acetylation is normalised, the acetylation signature is epiphenomenal	[118]
Monoaminylation-based accounts	E1 for the mark and for its weeks-long regulation in ventral tegmental area; never profiled after a psychedelic. H3Q5ser and related monoamine marks at cis-regulatory elements	Paired H3Q5ser and H3K27ac profiling at matched timepoints in the same nuclei. Persistence that tracks the monoaminylation mark while acetylation normalises relocates the substrate; loss of persistence after TGM2 manipulation, with acetylation intact, favours the monoaminylation account	[29,30]
Excitation/inhibition rebalancing	E2; not measured at persistence timepoints. Parvalbumin interneuron excitability	Loss of persistence with PV physiology intact and enhancer landscape displaced favours EL; the converse favours E/I	[119]
Learning-based accounts and ordinary consolidation	E1 for enhancer priming after ordinary experience. Behavioural trace; experience-dependent enhancer priming	Enhancer priming uncoupled from basal RNA also occurs in the hippocampal engram after ordinary experience, so persistence alone does not discriminate. Such an account requires a causal contribution to modified capacity for a subsequent experience, separable from retention of the initial one	[41,44]
Proteostatic persistence	E1 for the individual components; E2 as a substrate of psychedelic persistence. Long-lived scaffold pool (PSD-95, palmitoylation-locked)	Block PSD-95 palmitoylation and measure both readouts. Shortened persistence with the enhancer landscape still displaced demonstrates a scaffold requirement for expressing the state, not an alternative store (Section 4.4); unchanged persistence with a proteome shown equivalent to naive favours EL	[14,24,25,63,64]
Sustained sparse transcription	E2; inferred from the projection-specific behavioural requirement, not measured. Low-amplitude ongoing transcription in pyramidal tract neurons	Single-nucleus or projection-targeted RNA sequencing at seven days. Detectable sustained elevation in that class alone removes the premise that transcription has normalised, and with it the motivation for a chromatin store	[7]
Null account: ordinary consolidation	Not tested; the most parsimonious alternative available. No dedicated molecular store; enhancer changes are a correlate	Interrupt consolidation behaviourally (post-dose interference, environmental deprivation) with the enhancer landscape left intact and take the response to a subsequent plasticity challenge as the endpoint. Loss of the acquired increment in plastic capacity with displacement preserved favours the null account	[41,44]

## 8. Limitations and Unresolved Questions

The enhancer-latching account is a conceptual synthesis rather than a validated causal chain. The limitations of the evidence reviewed above are set out below without hedging.

Most mechanistically detailed data, including the persistent splicing changes [62] and the seven-day enhancer signature [11], come from preclinical rodent models, raising real questions of interspecies translatability and of divergent regulatory architecture in human cortex. The chromatin evidence rests substantially on a single dataset, and its limitations are better stated together than distributed across this review: one compound (DOI) at one dose, in one laboratory, in adult male mice only; bulk NeuN-positive nuclei rather than resolved cell types; a six-cluster partition whose cluster number was fixed by the analysts; no chromatin timepoint inside the acute window and none beyond seven days; no reported cluster-level test against vehicle at that final timepoint; and no reported time of day for tissue collection. What the wider literature supplies is convergent evidence for the phenomenon of transcriptome normalisation alongside a persistent phenotype, across compounds and species [14,19], rather than independent replication of the chromatin measurement itself. Combined with the scope restriction in Section 2.2, which places clinical durability data outside the reach of any substrate-level test, this means the account is at present testable only in rodents and only within a narrow window.

Although changes in H3K27ac are observed for seven days after administration [11], the causal necessity of these modifications has not been established by targeted intervention, and until the experiments of Test 2 are performed the mark remains a correlate. The 7-to-60-day bound stated in Section 2.3 is a prospective commitment, not an inference from data. Several propositions are sometimes attached to accounts of this kind but are not supported by the evidence reviewed here, and are listed so that they are not read in: an extrinsic anchor for the explanatory regress and a rate-versus-composition signature of a maintainer (Section 4.3), a serial glia-to-neuron chain (Section 5.3), intrinsic therapeutic or pathological valence of enhancers and occlusion at short dosing intervals (Section 6), and any extension to psychological-level accounts of belief revision (Section 4.5). One premise should also be recorded as a premise rather than as a finding: the temporal criterion that determines the form of the account rests on acetylation turnover measured in other systems and by bulk labelling [20,21,22], not in the neurons or at the elements at issue.

Three open problems are more serious than the rest. The first is the missing intercellular mediator set out in Section 5.3: the glial permissive branch and the neuronal enhancer branch cannot presently be joined into a serial chain, because the NF-κB-to-HDAC2 link on which such a chain would depend is characterised in cortical pyramidal neurons rather than in glia [69]. The second is that no candidate maintainer satisfies the three requirements of Section 4.3, so the literature currently names the problem of persistence more precisely than it solves it. The third is the resolution mismatch: the enhancer data are from bulk NeuN-positive nuclei [11], the splicing data are cell-type resolved [62], and the behavioural effect requires a specific projection class [7], so the three principal pillars are not matched in cellular resolution and cannot yet be aligned. None is fatal, none can be argued away, and all three should be resolved before the account is treated as more than a hypothesis.

Further constraints follow. The directional assignment proposed in Section 4.2 awaits the functional classification it presupposes, and that classification does not yet exist in an operationalised form. Describing the persistent sets as elevated and depressed rather than as poised is a terminological correction, but it also marks a genuine gap: the full histone signature of the persistent state, including histone H3 lysine 4 monomethylation (H3K4me1) and histone H3 lysine 27 trimethylation (H3K27me3), has not been profiled, and without it the chromatin state we invoke remains incompletely specified. Of the five set-point anchors proposed in Section 4.3, two are drawn from adjacent literatures rather than observed in this system [55,56], one is a metabolic inference, one rests on a motif enrichment rather than on a functional experiment, and the fifth—histone monoaminylation—has never been profiled after a psychedelic in any species [29,30]. The interplay between biological latching and psychological context—described through the relaxed-beliefs-under-psychedelics (REBUS) [120] and revised-beliefs-after-psychedelics (REBAS) [108] models—requires further investigation to determine whether molecular remodelling is a primary driver of, or a secondary response to, shifts in cognitive dynamics; both frameworks currently rest on small samples, and one of the supporting datasets is a preprint [107].

Finally, because a persistent cis-regulatory configuration is a candidate causal contributor to metaplasticity rather than an alternative account of it [40], the burden of proof it carries must be stated precisely. Demonstrating durable metaplasticity after psychedelic exposure would not establish it. In the pre-specified paradigm, verified absence of the candidate regulatory configuration during the window in which its contribution is predicted would reject that version of the account. The delimitation matters: a null result for H3K27ac would not exclude configurations defined by other chromatin properties, and failure to detect a signature is not the same as its verified absence, so the criterion applies only where the measurement has demonstrated sensitivity to the effect size at issue. Taken together, these constraints define the experimental programme this literature implies; the criteria of Section 2.5 and the discriminating observations of Table 3 are the appropriate near-term agenda.

### Translational Limitations

Three limits bear on clinical interpretation and are stated together. First, cross-compound reproducibility of the persistence measurement does not exist. The seven-day signature was obtained with a single compound (Section 4.1); the psilocybin enhancer dataset was collected on the day after administration and therefore characterises the acute response (Section 4.1); the persistent splicing data remain a preprint (Section 4.4); and the seven-day elevation of a presynaptic marker was measured at the protein level in pig, without a matched transcript or chromatin readout in the same animals (Section 4.4). What the wider literature supplies is convergent evidence for the phenomenon of transcriptome normalisation alongside a persistent phenotype across compounds and species, not replication of the chromatin measurement. Second, the regulatory architecture of human cortex differs from that of mouse at precisely the level at issue—enhancer sequence, enhancer–gene assignment and cell-type composition—so neither the loci nor the directions reported here transfer by assumption. Third, no substrate-level measurement is available in humans, and the scope restriction of Section 2.2 places clinical durability data outside the reach of any such test; the human paradigms cited there can corroborate or exclude the phenomenon but not the configuration proposed as its substrate. Before an enhancer-level mechanism could inform therapeutic development, three things would be required: replication of the persistence measurement with at least two structurally distinct compounds, cell-type-resolved data at timepoints beyond seven days, and a demonstration that the configuration contributes causally to re-inducible plasticity rather than accompanying it.

## 9. Conclusions

Read together, the evidence reviewed here reframes the persistence paradox as a question about state variables rather than about surviving molecules, within a bounded scope: the consolidation window of days to weeks, not the years of clinical follow-up. What the evidence currently supports is narrow. A persistent cis-regulatory configuration, resolved by cell type and by locus, could contribute causally to plastic responsiveness after psychedelic exposure; H3K27ac is a candidate indicator of that configuration—candidate also in the sense that its own relation to enhancer activity is experimentally contested; and the maintenance mechanism, the cis selector and the relation to circuit-level memory all remain to be determined.

What distinguishes this from a restatement of metaplasticity is the object of the claim. Persistent chromatin priming, dissociation from basal transcription and differential response at recall are documented in the hippocampal engram after ordinary experience, and in differentiated cells outside the nervous system. A pharmacological account therefore requires what ordinary-learning work has had no occasion to establish: that the configuration contribute causally to a modified capacity to respond to a subsequent experience, separable from retention of the experience already had.

Three quantities would make that demand decidable by experiment, and each is currently limited. The dominant relaxation constant for the persistent sets is stipulated in advance at approximately 7 to 60 days, and no existing datum licenses that range, because the time course ends at seven days and the persistently elevated set has not begun to relax. Of the six tests, four are attackable with established methods; the causal test remains a programme rather than an experiment, because a sign-respecting normalisation of thousands of elements exceeds the reach of targeted epigenome editing; and the comparison with the acute response cannot be attempted with existing data at all. And the three open problems set out in Section 8 stand before any of it.

Three experiments are the immediate priorities for the field, in order. Profile H3Q5ser and H3K27ac in the same nuclei at seven and thirty days after a single dose, with transglutaminase 2 manipulation, and ask whether persistence tracks the monoaminylation mark while acetylation normalises. Re-analyse the deposited chromatin data with cluster number fixed by an explicit criterion and test the chromatin–transcript dissociation locus by locus as an equivalence against a pre-specified margin rather than as a failure to reject the null. And sequence projection-targeted or single nuclei at seven days in pyramidal tract neurons, which tests the premise on which the whole framework rests: that sustained transcription has in fact normalised in the population that carries the behaviour.

The biological substrate of psychedelic metaplasticity therefore remains unresolved, and enhancer latching remains a proposed framework awaiting direct experimental validation of causality. What this review offers is not a resolved mechanism but a better-specified problem, together with the controls any solution must satisfy. Whether a persistent enhancer configuration is the physical store of psychedelic metaplasticity, or a necessary mediator of a state held elsewhere, is not decided by present evidence; stating that precisely, with the discriminating experiments attached, is more useful at this stage than an answer the data cannot support.

## Figures and Tables

**Table 1:** A systematic framework for empirical testing. Layers of the enhancer-latching account, with the timescale each layer would cover, the current evidentiary status of the supporting data, and the principal outstanding test associated with it. “Established” denotes peer-reviewed experimental demonstration; “preprint” denotes data not yet peer reviewed; “proposed” denotes an inference drawn in this review from the convergence of separate literatures. Throughout the text and in the tables that follow, these statuses are abbreviated as evidence levels: E1, established—peer-reviewed experimental demonstration in the system to which the claim is applied; E2, indirectly supported—peer-reviewed demonstration in a different system, cell type, compound or timescale and extended here by inference, or an association such as motif enrichment or functional annotation rather than a direct measurement; E3, hypothetical—an inference drawn in this review from the convergence of separate literatures, with no direct test. Preprint status is marked (pre) and is orthogonal to level. Abbreviations: 5-HT2A—serotonin 2A receptor; ACSS2—acetyl-CoA synthetase 2; BDNF—brain-derived neurotrophic factor; ECM—extracellular matrix; Egr—early growth response; Gq—Gq-family heterotrimeric G protein; GSK3β—glycogen synthase kinase 3β; H3K27ac—histone H3 lysine 27 acetylation; HDAC2—histone deacetylase 2; miRNA—microRNA; NeuN—neuronal nuclear antigen; NF-κB—nuclear factor κB; NFIL3—nuclear factor interleukin-3-regulated; PDZ—PSD-95/Dlg/ZO-1; PSD-95—postsynaptic density protein 95; PTM—post-translational modification; Rac1—Ras-related C3 botulinum toxin substrate 1; -SCV—serine–cysteine–valine; TGM2—transglutaminase 2; TrkB—tropomyosin receptor kinase B.

Layer	Proposed Timescale	Evidentiary Status	Principal Outstanding Test	Refs.
Intracellular 5-HT2A trigger (Golgi pool; location bias)	Minutes–hours	E1/E3—Established for location bias; Golgi-restricted TGM2/Rac1 serotonylation proposed here	Compartment-restricted TGM2 inhibition should abolish psychedelic-induced spinogenesis without affecting acute Gq signalling	[9,46]
PSD-95 receptosome and Kalirin-7/Rac1 spinogenesis	Minutes–days	E1—Established	Disruption of the -SCV/PDZ interaction should uncouple acute signalling from durable spine gain	[10,47,48,49]
5-HT2A–TrkB convergence	Hours–days	E2, contested—Functional convergence supported, system- and output-dependent; obligate physical complex not demonstrated; direct kinase binding contested	An intervention separating cooperation, permissive dependence and obligate physical association; the enhancer-level account is unaffected by the outcome	[2,50,51]
Bidirectional enhancer displacement (H3K27ac)	Days (≥7 days)	E1—Established, bulk NeuN+ resolution, single primary dataset; additional sex- and region-dependent evidence, collected 24 h after psilocybin and therefore not addressing persistence	Functional sign of the depressed set is undetermined; the permissive reading requires verified enhancer–gene links and measured re-inducibility	[11,52,53,54]
Maintenance of the displaced state (nucleosome turnover; acetyl-CoA/ACSS2; histone monoaminylation; ECM; circadian)	Days–weeks	E3—Proposed; no candidate satisfies all three requirements—identifiable variable, slow dynamics, verified coupling	Selective local normalisation with maintenance inputs intact should abolish the acquired responsiveness; targeted restoration should recover it	[29,30,36,55,56,57,58,59,60]
Circadian contribution to maintenance	Days–weeks	E2—Motif enrichment only; occupancy unmeasured; NFIL3 is sequence-specific and can repress	With mean, phase and amplitude separated, cycle disruption should shorten expression of the altered capacity without altering the acute response	[11,59,61]
Cell-type-specific alternative splicing	Weeks (≥1 month)	E2 (pre)—Preprint; single unreplicated dataset	Independent replication with cell-type resolution; loss of the splicing signature should track loss of persistence	[62]
Synaptic proteome and scaffold stabilisation (PTMs, miRNA)	Days–weeks	E1—Established for individual components; treated here as a competing account, not a subordinate layer	Blocking PSD-95 palmitoylation should shorten the persistence window; loss of the phenotype with the enhancer landscape unchanged demonstrates a scaffold requirement for expressing the state and does not by itself identify an alternative store (Section 4.4)	[14,24,25,63,64,65]
Glial permissive branch (NF-κB; GSK3β/HDAC2)	Days–weeks	E2/E3—Links individually supported; no serial glia-to-neuron chain is available, since the NF-κB–HDAC2 link is characterised in cortical pyramidal neurons rather than in glia	Identification of the intercellular mediator; factorial drug-by-manipulation design distinguishing permissiveness from instruction	[66,67,68,69,70,71]
ECM viscoelasticity and mechanotransduction to chromatin	Weeks	E2—Established in non-neuronal systems; extension to cortex proposed here	Pharmacological stiffening of the perineuronal ECM should reduce enhancer persistence	[55,72]
Therapeutic versus pathological latching	Days–weeks	E3—No exclusive locus-valence classifier is supported; BDNF–TrkB loci also serve reward	Identification of the cis selector; combinatorial selection is plausible but the psychedelic instance is unidentified	[73,74,75,76]

**Table 2:** The five candidate maintainers of a displaced set-point, assessed against the three requirements stated in this section. No candidate currently satisfies all three. Entries restate Section 4.3 and introduce no evidence beyond it; references are given in the corresponding text. Abbreviations: ACSS2—acetyl-CoA synthetase 2; H3.3—histone H3 variant 3.3; H3K27ac—histone H3 lysine 27 acetylation; H3Q5ser—histone H3 glutamine 5 serotonylation; NFIL3—nuclear factor interleukin-3-regulated; TGM2—transglutaminase 2.

Candidate	Identifiable Physical Variable?	Dynamics Slow Enough to Span the Interval?	Coupling to the Enhancer Configuration Verified?	What Would Have to Be Measured
Nucleosome turnover (H3.3 exchange)	Yes—the rate of exchange rather than the occupancy	Plausible	No	Exchange rate at the persistent enhancer set
Local acetyl-CoA availability (ACSS2)	Yes—local metabolite supply, which can carry pattern as well as duration	Plausible	No	Acetyl-CoA supply and ACSS2 occupancy at the persistent set after psychedelic exposure
Viscoelastic extracellular matrix	Weak as applied to cortex; characterised principally in fibroblasts and in reprogramming	Yes—remodelled within 24–48 h	No	Perineuronal matrix mechanics in adult cortex and their coupling to cis-regulatory state
Circadian gating (NFIL3)	Partly; a light–dark cycle shared by treated and control animals cannot by itself supply the address and sign of what is maintained	Yes	No—motif enrichment only, occupancy unmeasured	NFIL3 occupancy, with change in mean, phase and amplitude separated
Histone monoaminylation (H3Q5ser; TGM2)	Yes	Unknown, and directly measurable by the approaches used for acetylation	No—H3Q5ser has not been profiled after a psychedelic in any species	H3Q5ser and H3K27ac in the same nuclei, with TGM2 manipulation

## Data Availability

No new data were created or analysed in this study. Data sharing is not applicable to this article.

## Abbreviations

The following abbreviations are used in this manuscript:

25CN-NBOH	*N*-(2-hydroxybenzyl)-2,5-dimethoxy-4-cyanophenylethylamine
5-HT	Serotonin
5-HT2A	Serotonin 2A receptor
5hmC	5-hydroxymethylcytosine
5mC	5-methylcytosine
ACSS2	Acetyl-CoA synthetase 2
AhR	Aryl hydrocarbon receptor
Akt	Protein kinase B
Arc	Activity-regulated cytoskeleton-associated protein
BDNF	Brain-derived neurotrophic factor
CaMKII	Ca^2+^/calmodulin-dependent protein kinase II
cAMP	Cyclic adenosine monophosphate
CARC	Cholesterol recognition motif
CBP	CREB-binding protein
CREB	cAMP response element-binding protein
DAG	Diacylglycerol
DMT	*N*,*N*-dimethyltryptamine
DOI	2,5-dimethoxy-4-iodoamphetamine
DOM	2,5-dimethoxy-4-methylamphetamine
E/I	Excitation/inhibition
ECM	Extracellular matrix
Egr	Early growth response
EL	Enhancer latching
ERK	Extracellular signal-regulated kinase
FACS	Fluorescence-activated cell sorting
GSK3β	Glycogen synthase kinase 3β
GTP	Guanosine triphosphate
H3K4me1	Histone H3 lysine 4 monomethylation
H3K4me3	Histone H3 lysine 4 trimethylation
H3K27ac	Histone H3 lysine 27 acetylation
H3K27me3	Histone H3 lysine 27 trimethylation
H3Q5ser	Histone H3 glutamine 5 serotonylation
HDAC	Histone deacetylase
HDAC2	Histone deacetylase 2
HIRA	Histone cell cycle regulator
IP3	Inositol trisphosphate
LSD	Lysergic acid diethylamide
LTP	Long-term potentiation
MAGI	Membrane-associated guanylate kinase with inverted orientation
MAGUK	Membrane-associated guanylate kinase
MAPK	Mitogen-activated protein kinase
MDMA	3,4-methylenedioxymethamphetamine
MEK	Mitogen-activated protein kinase kinase
miRNA	MicroRNA
mTOR	Mammalian target of rapamycin
NeuN	Neuronal nuclear antigen
NF-κB	Nuclear factor κB
NFIL3	Nuclear factor interleukin-3-regulated
NMDA	*N*-methyl-D-aspartate
PAK	p21-activated kinase
PAK1	p21-activated kinase 1
PAR	Proline- and acidic-amino-acid-rich
PDZ	PSD-95/Dlg/ZO-1 domain
PFC	Prefrontal cortex
PI3K	Phosphoinositide 3-kinase
PIP2	Phosphatidylinositol 4,5-bisphosphate
PKC	Protein kinase C
PLCβ3	Phospholipase C-β3
PSD-95	Postsynaptic density protein 95
PTM	Post-translational modification
PV	Parvalbumin
Rac1	Ras-related C3 botulinum toxin substrate 1
REBAS	Revised beliefs after psychedelics
REBUS	Relaxed beliefs under psychedelics
RSK2	Ribosomal S6 kinase 2
SCV	Serine–cysteine–valine motif
SERT	Serotonin transporter
TFIID	Transcription factor II D
TGM2	Transglutaminase 2
TrkB	Tropomyosin receptor kinase B
